# Linear and branched *β-*Glucans degrading enzymes from versatile *Bacteroides uniformis* JCM 13288^T^ and their roles in cooperation with gut bacteria

**DOI:** 10.1080/19490976.2020.1826761

**Published:** 2020-10-10

**Authors:** Ravindra Pal Singh, Sivasubramanian Rajarammohan, Raksha Thakur, Mohsin Hassan

**Affiliations:** a Food and Nutrition Biotechnology Division, National Agri-Food Biotechnology Institute, Mohali, India; b Agricultural Biotechnology Division, National Agri-Food Biotechnology Institute (NABI), Mohali, India

**Keywords:** Enzymes, glycan utilization, macroalgae, gut bacteria, cross-feeding

## Abstract

*β-*glucans are the dietary nutrients present in oats, barley, algae, and mushrooms. The macromolecules are well known for their immune-modulatory activity; however, how the human gut bacteria digest them is vaguely understood. In this study, *Bacteroides uniformis* JCM 13288 ^T^ was found to grow on laminarin, pustulan, and porphyran. We sequenced the genome of the strain, which was about 5.05 megabase pairs and contained 4868 protein-coding genes. On the basis of growth patterns of the bacterium, two putative polysaccharide utilization loci for *β-*glucans were identified from the genome, and associated four putative genes were cloned, expressed, purified, and characterized. Three glycoside hydrolases (GHs) that were endo-acting enzymes (*Bu*GH16, *Bu*GH30, and *Bu*GH158), and one which was an exo-acting (*Bu*GH3) enzyme. The *Bu*GH3, *Bu*GH16, and *Bu*GH158 can cleave linear exo/endo- *β-* 1-3 linkages while *Bu*GH30 can digest endo- *β-* 1-6 linkages. *Bu*GH30 and *Bu*GH158 were further explored for their roles in digesting *β-* glucans and generation of oligosaccharides, respectively. The *Bu*GH30 predominately found to cleave long chain *β-* 1-6 linked glucans, and obtained final product was gentiobiose. The *Bu*GH158 used for producing oligosaccharides varying from degree of polymerization 2 to 7 from soluble curdlan. We demonstrated that these oligosaccharides can be utilized by gut bacteria, which either did not grow or poorly grew on laminarin. Thus, *B. uniformis* JCM 13288 ^T^ is not only capable of utilizing *β-*glucans but also shares these glycans with human gut bacteria for potentially maintaining the gut microbial homeostasis.

## Introduction

Human gut microbiota is composed of about 10^13^–10^14^ microbial cells with an estimate of 5000 bacterial species.^1^ These bacterial species predominantly belong to *Firmicutes, Bacteroidetes, Actinobacteria*, and *Proteobacteria*.^2^ These commensal bacteria provide several benefits to the host by harvesting energy, regulating host immunity, protecting against pathogens, and maintaining gut homeostasis by microbial cooperation.^3,4^ These communities mainly provide health benefits in two ways; thriving on dietary nutrients and producing valuable metabolites, such as short-chain fatty acids (SCFAs).^5^ Thriving on dietary nutrients by bacterial communities depends on their capabilities to produce carbohydrate-active enzymes (CAZymes). These enzymes are highly diverse and are commonly absent in the human genome; thus, genes of bacterial communities are often represented as the secondary genome of the host.^6^ Gram-negative bacteria (such as *Bacteroidetes*) possess a wide variety of glycoside hydrolases and polysaccharide lyases, whereas Gram-positive bacteria (such as *Lactobacillus* and *Bifidobacteria*) mainly possess glycoside hydrolases for digesting variety of dietary fiber.^2,7^ These CAZymes are co-ordinately present in *Bacteroides* in so-called polysaccharide utilization loci (PULs). PULs consist of clustered and co-regulated genes that encode enzymes, glycan-utilizing transporters, and sensor proteins, which control the transcription of the cognate locus upon the availability of specific nutrients.^8,9^ Until now, only a few PULs have been biochemically characterized in *Bacteroidetes*,^2^ despite thousands of them being identified.^7^

The *β-* glucans have gained strong attention as an imperative in food supplements, wherein they can act as either immunostimulants in cancer treatments and inflammation^10,11^ or microbiome modulatory agents.^12^ The *β-* glucans are predominantly present in the daily human diet in soluble and insoluble fiber states. These are structurally diverse with a variety of glycosidic linkages. For instance, *β-*glucans extracted from *Euglena* (known as paramylon) and bacterial polysaccharides (known as curdlan) are linear in chain with *β-*1-3 linked,^13^ while marine macroalgal-extracted glucan (known as laminarin) is branched with *β-*1-3 and *β-*1-6 linkages.^14^ Furthermore, frequency and length of *β-*1-6 linked glucans depend on the type of sources, i.e. yeast, *Laminarin digitata, Lasallia pustulata*, and *Lentinus edodes*.^15–18^ Curdlan is one of the abundant bio-resources that can be synthesized by several bacteria, including *Agrobacterium*,^19^ *Rhizobium*,^20^ and *Cellulomonas* species.^21,22^ In addition, huge quantities of laminarin can accumulate in marine environment upon degradation of macroalgae, and plays a major role in marine carbon cycle.^23,24^ Therefore, extraction of curdlan and laminarin is much simpler as compared to other natural glycans and can be easily exploited for nutraceutical perspectives.^25–27^ However, laminarin and curdlan utilizing capability of gut bacteria is still poorly understood.

Utilization of *β-*1,3-glucan involves the actions of *β-*1,3 glucanases (EC 3.2.1.6 and EC 3.2.1.39) and *β-* 1,3 glucosidase (EC 3.2.1.58) that belong to the glycoside hydrolase families, GH5, GH16, GH17, GH55, GH64, GH81, GH128, and GH158, and GH 3, 5, 17, and 55, respectively.^28–33^ The *β-*1,3-glucanases cleave internal glycoside bond of the substrate and produce oligosaccharides, whereas *β-*1,3-glucosidases act on non-reducing ends of *β-*1,3-glucans and release glucose from the generated oligosaccharides.^34^ Some of the endo-acting *β-*1,3-glucanases can have carbohydrate-binding domains for improving the capability of those enzymes to bind water-insoluble substrates.^35,36^ The utilization of branched chain of *β-*1,6 linked-glucan requires *β-*1,6-glucanase (EC 3.2.1.75), belonging to the GH30 family. A *β-*1,6-glucanase has been reported in *Bacteroides thetaiotaomicron* that cleaves endo-1,6 linkages on long chain of linked glucose residues present in the fungal cell wall.^37^ In case of laminarin, there is a *β-*1,6 linked glucose unit, and an exo-acting enzyme (*β-*1,6-glucosidase) is required to efficiently utilize the glycan along with *β-*1,3 glucanase as identified in marine bacteria so far.^23,24^

In this study, we sequenced the genome of the human gut commensal *Bacteroides uniformis* JCM 13288 ^T^ that was shown to grow on several *β-* glycans. Subsequently, we identified two PULs by comparative genomic analysis that are involved in the utilization of different *β-* glucans. We also did comparison of synteny of these PULs with other *Bacteroides*, and integrative conjugative element (ICE) with *Bacteroides plebeius* DSM 17135. A detailed kinetics and characterization of PUL associated *Bu*GH158 and *Bu*GH30 were performed. Occasionally, bacterial PULs associated endo-acting enzymes generate several oligosaccharides with varying degree of polymerization (DPs) 2 to 7 from soluble curdlan, which are found to be involved in cross-feeding with other gut beneficial bacterial communities.^4^ Those cross feeders do not encode a gene for endo-acting enzymes but can utilize oligosaccharides. In order to understand role of identified PUL in cross-feeding, the *Bu*GH158 was further exploited for generating *β-*1, 3-oligosaccharides (*β-*1, 3-OSs) that were purified and characterized. Subsequently, we demonstrated that the generated *β-*1, 3-OSs can allow other human gut bacteria to grow on this substrate.

## Materials and methods

### Materials and chemicals

Gentiobiose, low melting point agarose, and laminarin from *Laminaria digitata* were purchased from Sigma Aldrich (Germany). Curdlan from *Alcaligenes faecalis*, lichenan (also known as lichenin) from Icelandic moss, yeast *β-* glucan, laminaribiose and laminaripentaose were purchased from Megazyme, Ireland. Pustulan from *Lasallia pustulata*, and porphyran from *Porphyra* were purchased from Elicityl, France and Carbosynth, United Kingdom, respectively. Used bacterial strains were purchased from the Japan Culture Collection, Riken, Japan.^1^H NMR, DEPT135, and 2D NMR (COSY and HSQC) analyses were recorded at the Panjab University, Chandigarh, India.

### Genome sequencing and annotation

Genomic DNA from *B. uniformis* JCM 13288 ^T^ was isolated using GenElute™ bacterial genomic DNA Kit (Sigma Aldrich, NA2110-1KT). Purity of the genomic DNA was first checked on 0.8% agarose gel and NanoDrop One spectrophotometer (ThermoFisher Scientific), and then the genomic DNA was further purified using 0.45X Ampure XP magnetic beads to remove other contaminants in the DNA preparation. Genomic DNA was sequenced using the Nanopore minION system. About 3 µg of high molecular weight purified genomic DNA was used for library preparation using the ligation sequencing kit (SQK-LSK109), and following ONT ligation protocol as per manufacturer’s instructions with minor modifications. About 800 ng of the prepared libraries were loaded and run on R9.4 SpotON MinION flow cells for 4 h with live base-calling enabled. The MinION run produced 85542 reads, amounting to ~770 Mb of data resulting in approximately 140 x coverage of the genome. The genome was assembled using the Canu assembler version 1.9.^38^ The assembly was further polished to generate a consensus sequence using medaka version 1.0.3. The assembly thus generated was deposited in NCBI under the BioProject ID PRJNA636247.

The genome was annotated using the Prokka annotation pipeline. The ORFs were predicted using Prodigal (built-in software in Prokka).^39^ The sequences were then queried against the default databases – Uniprot and NCBI-nr database, using DIAMOND blastp.^40^ The best significant match with an e-value of ≤ 10^−10^ was used to annotate the gene products/proteins. Genes coding for CAZymes were identified following a BLAST search against the CAZy database using a threshold of e-value ≤ 10^−10^. Each gene entry in the genome was manually curated and annotated by combining the annotation results across all databases. Putative polysaccharide utilization loci (PULs) were identified by finding all SusC (TonB-dependent receptor)- or SusD (outer membrane glycan-binding protein)-like protein-coding genes. The locus was labeled as a PUL if it contained at least two or more CAZymes within ±5 kb of the SusC- or SusD-like protein-coding genes.

### Assessment of bacterial growth on laminarin, porphyran, and low melting point agarose

Active culture of *Bacteroides uniformis* JCM 13288 ^T^ was initially made by inoculating 5 µL of glycerol stock into 10 mL of freshly prepared Gifu anaerobic medium (GAM broth) at 37°C. Anaerobic condition during bacterial growth performance in an anaerobic jar was maintained via filling anaerobic gas (20% CO_2_ and 80% H_2_) using an Anoxomat machine. Once strains were grown, bacterial cells were centrifuged and washed with phosphate-buffered saline (PBS) before their growth assessment performed in 200 μL of minimal medium containing 1% laminarin, low melting point agarose, λ carrageenan, pustulan, or porphyran on a 96-well plate, following our recently established method.^41^ The growth assessment experiments were carried out for 72 h in anaerobic condition at 37°C, where growth was observed every alternative 12 h at 600 nm using an ELISA plate reader. The experiment was repeated at least three times to confirm the observations.

### Cloning, expression, and purification of enzymes

**Figure 1.**
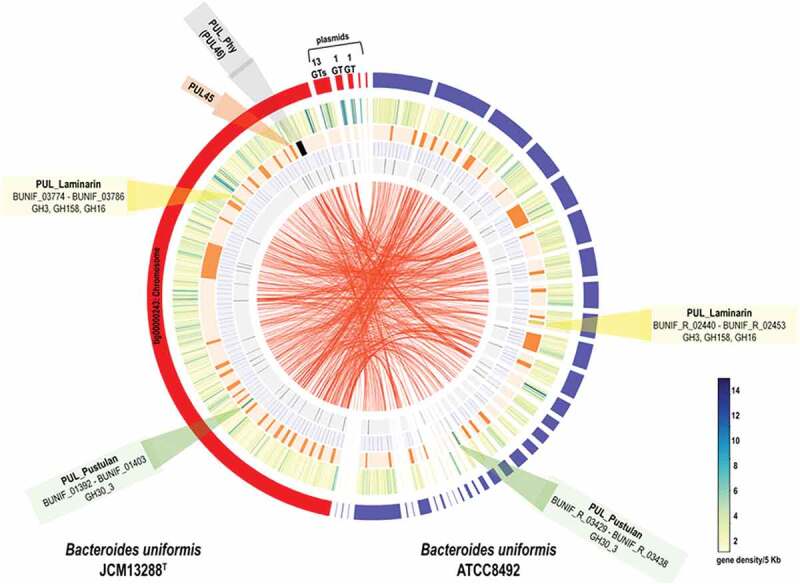
A comparative genome analysis of the *B. uniformis* JCM13288^T^ with the *B. uniformis* JCM 5828 (ATCC 8492). The tracks (outside to inside) represent (i) density of protein-coding genes per 5 kb, (ii) location of PULs, (iii) CAZymes, (iv) transposases and (v) the synteny between the two genomes. The PULs responsible for laminarin utilization are marked in yellow (GH3, GH158, and GH16) and green (GH30_3) and the PUL responsible for porphyran is marked in black. The *B. uniformis* JCM13288^T^ has five plasmid-like sequences that contain genes for several glycosyl transferases.

**Figure 2.**
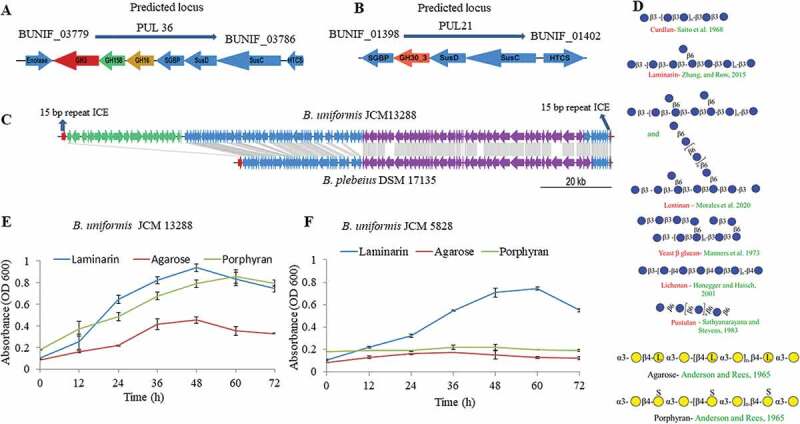
Annotated polysaccharide utilization locus (PUL) and bacterial growth patterns. Illustration of laminarin, fungal glucans and agarose- porphyran utilization genes orientation with their relative sizes (A, B, and C). (c) Comparison of synteny and integrative conjugative element of the *B. uniformis* JCM13288^T^ and *Bacteroides plebeius* DSM 17135, highlighting the presence of extra PUL (PUL45) adjacent to porphyran utilization locus without disrupting its function. (d) schematic representation of several glycans used in the study. (e and f) growth patterns of both *Bacteroides uniformis* strain on different substrates. Three independent replicates were used for growth performance. Graph represent average value of three independent replicates with standard errors.

Predicted laminarin utilization loci were identified in genome of the *B. uniformis* JCM 13288 ^T^ and putative laminarin utilizing enzymatic genes were identified as BUNIF_03780, BUNIF_03781, BUNIF_03782 and BUNIF_01399 (Figures 1 and 2(a and b)) as well as BPGH30 (gene ID- 2515950412) from the *Blautia producta* JCM 1471 ^T^. First of all, signal peptides of these genes were predicted using TMHMM Server, v. 2.0, and SignalP, v. 4.1.^42^ Thereafter, set of primers were designed after excluding signal peptide and lapidated amino acids region,^43^ and restriction sites were carefully selected after examining that there were no restriction sites within the gene (Table S1). PCR reaction mixture (100 μl) contained of 10 μl of 10 × PCR buffer containing MgCl_2_, 25 mM of each deoxynucleotide triphosphate (dATP, dCTP, dGTP, and dTTP), 100 ng of each primer, 100 ng of purified DNA template, and 1 unit of PfuUltra high fidelity DNA polymerase (Agilent). The PCR run cycles included a 5 min initial denaturation at 95°C, followed by 30 cycles at 95°C for 45s, 68°C or suitable Tm for 45s and 72°C for 1–3 min, with a final cycle of 5 min at 72°C. PCR products were purified through GeneJET™ gel extraction kit (k0691), and quality was checked on 1% agarose gel (Fig. S1). Finally, genes were cloned into vector pET28a (Novagen) with N-terminal (His)_6_-tag. Cloned constructs were first transformed into *Escherichia coli* TOP10 cells, and then plasmid preparation was carried out to check genetic integrity of all desired inserts (Supplementary Fig. S1). Subsequently, the cloned constructs were transformed into *E. coli* expression cells, BL21 (DE3).

*E. coli* BL21 cells were cultured in Luria-Bertani (LB) or Terrific broth medium containing kanamycin (50 µg/ml) to mid-log phase (A_600_ nm of ~0.6) or OD ~ 1.2 at 37°C, respectively. Afterward, protein expression was induced by adding 0.5 mM isopropyl *β-*D-1-thiogalactopyranoside (IPTG) to the cell cultures and further incubated at 22°C for 16 h. Post incubation, cells were collected by centrifugation at 4,000 x g and re-suspended in 50 mM HEPES buffer containing appropriate EDTA-free protease inhibitor cocktail (Sigma Aldrich, Cat. No. 4693159001). Re-suspended cells were disrupted by ultra-sonication and supernatant was collected by centrifugation (16,000 x *g*) for 20 min at 4°C. All four proteins were purified from supernatant by immobilized metal affinity chromatography (IMAC) using nickel-nitrilotriacetate (Ni-NTA) – agarose-based matrix, applying gradient of imidazole (10, 50, 100, 150, 200, and 500 mM) containing 500 mM NaCl. Desired proteins were eluted in 50–100 mM imidazole containing fractions, and then imidazole was replaced with 50 mM HEPES buffer by Amicon® Ultra-15 Centrifugal Filter Unit (10 kDa). If required, further purification of protein was performed with Superose^TM^ 6 Increase 10/300 GL column on ÄKTA protein purification system. A 10% sodium dodecyl sulfate–polyacrylamide gel electrophoresis (SDS-PAGE) was used for determining molecular weight of the desired proteins. Concentration of purified enzymes was finally determined by spectrophotometer using Bradford reagent, and stored at −80°C, having concentration of 1 mg/ml for further use.

### Evaluating biochemical properties of enzymes

Enzymatic assays were performed in 100 µl reaction mixture containing 10 µl enzyme (1 mg/ml) and 10 µl of 1% substrate (such as curdlan, laminarin, or yeast *β-* glucan) in optimized buffer. Released reducing sugars by activity of enzyme were determined using 3,5- dinitrosalicylic acid (DNS) assay.^44^ In brief, after appropriate time of incubation (2 h) of enzymatic reactions, 80 µl of DNS reagent was added into reaction mixture and then incubated at 100°C for 10 min, followed by chilling on ice for 10 min. Subsequently, reaction mixture was centrifuged at 10000 × g for 10 min to remove the undigested substrate, and then reading of supernatant was recorded at 540 nm spectrophotometrically. Amounts of released reducing sugars were finally determined by standard curve, generated using different concentrations of glucose.

Optimum pH of different enzymes was determined in different buffers by incubating reaction at 37°C for 2 h in a standard reaction cocktail. Different buffers such as phosphate – citrate (pH 3 to 5), sodium – phosphate (pH 6 and 7) and HEPES (pH to 7.4 to 9) were used at 50 mM. For determining optimal temperature, standard reactions (100 µl) having 10 µl enzyme (1 mg/ml) and 10 µl of 1% suitable substrate were performed using optimized pH at different temperatures (22, 30, 37, 45, and 54°C) for 2 h.

Michaelis Menten parameters of each enzyme in terms of Km, Vmax and Kcat were determined using various concentrations of different substrates, such as laminarin, curdlan, lichenan, and lentinan. Different concentrations of these substrates, ranging from 0.001 to 20 mg/ml were used for kinetics parameters with suitable concentration of enzyme in 50 mM sodium – phosphate buffer (pH 7.0) at 37°C for 2 h of incubation. Afterward, reaction was stopped by incubating at 100°C for 5 minutes. Enzymatic assays with these substrates were determined with DNS assays, as above mentioned,^44^ and thin-layer chromatography (TLC). TLC analysis was carried out on Silica Gel 60 F254 (Merck) and generated mono- and oligo-saccharides were visualized by spraying TLC with 5% H_2_SO_4_ in ethanol, followed by charring. Kinetics parameters such as K_m_, V_max_, and K_cat_ were analyzed on GraphPad Prism software. Three independent tests were performed for each experiment.

### Pre-treatment of curdlan for improving hydrolysis potential of BuGH158

Many enzymes, that cleave curdlan, have previously shown retarded activity on crude substrate.^45,46^ Curdlan is a large molecular weight polysaccharide and can exist in a triple helix form due to inter and intra-molecular hydrogen bonding. Triple helix form of curdlan can easily be ionized by a dilute alkali solution, which coverts curdlan into singular form.^47^ Therefore, curdlan was pre-treated using two different methods. In the first treatment, 500 mg of curdlan was dissolved in 50 ml of 1% NaOH (pH 11.0) and then vigorously stirred for 30 min at 22°C, followed by neutralized the pH using acetic acid (1.5 M) – we named this as curdlan-A.^48^ In the second treatment, 1 g of curdlan was suspended in 50 ml of glycine-NaOH buffer (100 mM, pH 10.5) and vigorously mixed by a magnetic stirrer for 30 min at 22°C. After that the solution was neutralized by 0.1 N HCl and precipitant was collected after centrifugation (5000 × g) for 20 min. Pellet was again dissolved in 25 ml of milli-Q and heated for 70°C for 2 h, and precipitant was recovered through centrifugation (5000 × g) for 20 min, which was considered as substrate for enzymatic assay, we named this curdlan-B.

### Production, purification, and characterization of β-1, 3-OSs from curdlan

We exploited the *Bu*GH158 for production of *β-*1, 3-OSs from curdlan. Initial enzymatic reactions (200 µl) were performed on 50 mg of curdlan-A in Tris-HCl/HEPES buffer (pH 7.4) using 20 µl enzyme (1 mg/ml) for different time of periods (1 to 5 and 16 h). After that, several reactions were performed by taking different concentrations of curdlan-A (5 fold higher and lower of enzyme concentration) in 500 µl reaction mixture in Tris-HCl buffer (pH 7.4) by incubating reaction at 37°C for 1 to 5 h. Produced *β-*1, 3-OSs from enzymatic reaction mixture was recovered from undigested residues by centrifugation (5000 × g) for 10 min and then purified by Toyopearl S40 resin containing column (GE Healthcare- XK 26/100). All fractions were initially checked with TLC and same pattern fractions were pooled together and analyzed by MALDI-TOF.

MALDI-TOF mass spectrum was recorded in positive ion mode on purified *β-*1, 3-OSs, which was recorded using an AB SCIEX 5800 mass spectrometer. 2 μl sample (10 mg/ml solution) was mixed with double volume of 2,5-dihydroxybenzoic acid (30% acetonitrile) and dried on a MALDI target plate. Spectra from 200 laser shots were summarized to create a mass spectrum. A 5500 QTRAP mass spectrometer (AB Sciex, Foster City, CA, USA) was also used to determine the molecular mass (MS) of enzymatic products through direct injection method using electrospray ionization (ESI) source.

^1^H, ^13^C-DEPT- and 2D NMR of freeze-dried *β-*1, 3-OSs were performed on purified products generated from curdlan on Bruker Avance Neo 500 MHz spectrometer equipped with a broadband BBFO probe. Chemical shifts (*δ*) were demarcated in parts per million (ppm) in which residual solvent signal was defined as a reference point for assigning peaks of the sample. MestreNova software was used for data processing and analysis.

### Evaluating glycan cooperation between different human gut bacteria

In order to understand the role of generated *β-*1, 3-OSs on growth performance of Gram-positive probiotics bacteria, *Blautia producta* JCM 1471 ^T^, *Ruminococcus faecis* JCM 15917 ^T^, *Bifidobacterium pseudocatenulatum* JCM 1200 ^T^, *Bifidobacterium adolescentis* JCM 1275 ^T^ and *Anaerostipes caccae* JCM 13470 ^T^ were used in mono and co-culture experiments. Initially, culture of these bacteria was prepared in the same manner as mentioned above. For the determination of bacterial cooperation on agar petri-plates, 5 μl of the PBS suspended bacterial culture was dotted 5–8 mm adjacent to one another onto laminarin minimal medium agar petri-plates, along with control volume of PBS onto another plate. After 5 days of plate incubation in anaerobic condition, dotted areas were cut out from plates, diluted with PBS, and plated onto Gifu anaerobic agar petri-plates for CFU enumeration. Bacterial growth determination on generated *β-*1, 3-OSs was also performed following a protocol reported recently.^41^ Results of these analyses were statistically analyzed through t-test (significance level at *p* < .05).

## Results

### Genome sequencing, annotation, and glycobiome analysis

The genome of *B. uniformis* JCM 13288 ^T^ was sequenced and assembled using the Nanopore MinION sequencer and the Canu assembler. The final genome assembly consisted of a single contig of 5,055,696 bp representing the complete circular genome of *B. uniformis* and five putative plasmid sequences (Figure 1). A total of 4938 genes were annotated using a combination of Prokka and other databases including Uniprot, NCBI-nr and CAZy databases. Out of the 4938 genes in the genome, 4868 were protein-coding genes and 70 tRNA sequences. We identified 287 GHs, 14 polysaccharide lyases (PLs), 24 carbohydrate esterases (CEs), 187 glycosyltransferases (GTs), and 44 carbohydrate-binding modules (CBMs) annotated using the CAZy database (denoted as white track in Figure 1). A total of 46 PULs (denoted as plain orange track in Figure 1) were identified in the strain using the pipeline described in materials and method, all PULs are mentioned in supplementary excel sheet 1.

Similarly, we also re-annotated the assembly of reference strain of the *B. uniformis* JCM 5828 (ATCC 8492) to rule out differences in gene content due to the annotation pipeline used. We identified a total of 3932 genes out of which 3877 were protein-coding genes, and 55 were tRNA-coding sequences. The *B. uniformis* JCM 5828 genome encoded 256 GHs, 179 GTs, 20 CEs, 4 PLs, and 44 CBMs. Additionally, a total of 39 PULs were identified in the *B. uniformis* JCM 5828 genome using the same parameters as for the JCM13288^T^ strain. The PULs responsible for laminarin and pustulan utilization are marked in yellow (GH3, GH158, and GH16) and green (GH30_3) respectively, in Figure 1 and sketched in Figure 2(a and b).

Indeed, we have found a canonical PUL in *B. uniformis* JCM 13288 ^T^ that is corresponding to a locus present in marine bacterium, *Bacteroides plebeius* (Figure 2(c)). This locus (PUL46) may be responsible for agarose and porpharan saccharification (marked in black in Figure 1), which is missing in the reference strain *B. uniformis* JCM 5828. Since *B. uniformis* JCM 13288 ^T^ was isolated from adult Japanese gut, we predicted that the strain might have other marine glycan utilization loci, and surprisingly found a laminarin-PUL. The locus was conserved in both the *B. uniformis* strains (Figure 1). Therefore, it can be hypothesized that carbohydrate utilization potential of these strains is varied, as is evidenced by the differences in CAZyme, and PUL abundances.

### Screening with macroalgal and fungal glycans, identification of PULs and bioinformatics analysis

Both the strains did show robust growth on laminarin, while only *B. uniformis* JCM 13288 ^T^ showed strong and weak growth on porphyran and agarose, respectively (Figure 2(e and f)). There is a subtle difference in the structures of porphyran and agarose (Figure 2(d)). None of the strains were found to be growing on λ carrageenan (data not shown). Putative agarose – porpharan utilization locus (AP-PUL) contained 14 GHs (three GH16, four GH2, two GH86, each of GH117, GH50, GH105, GH3, and GH29). In addition, the locus contained several enzymes that could be involved in the metabolism of degraded products of agarose or porphyran in the cytoplasm, such as alcohol dehydrogenase (BUNIF_04673), aldo/keto reductase (BUNIF_04653) and L-galactose mutarotase (BUNIF_04654), which are predicted to be cytoplasmically localized.^49^ Homology searches of these amino acid sequences via the Joint Genome Institute Integrated Microbial Genomes and Metagenomes (JGI-IMG) revealed 100% similarity to genes present within *P*-PUL (the PUL has 12 GHs) of the *Bacteroides plebeius* DSM 17135 (Figure 2). Strikingly, all 42 proteins identified in putative AP-PUL found to be 100% identical to genes present in the genome of the *B. plebeius* DSM 17135. Three GH2, namely GH2A, GH2B, and GH2C (Supplementary excel sheet 1), showed homology to GH2 (*β-*galactosidase) of the *Vibrio sp*. EJY3 with 32, 46, and 35% sequence similarity, respectively, via JGI-IMG-blastP. These enzymes are likely to remove galactose from galactose *β-*1-4- 3,6 anhydro-D-galactose. Another rare exo-acting *β-*galactosidase (GH117) is also expected to work with GH2A/B/C to convert agarose into monosaccharides, which subsequently internalize into cytoplasm via RhaT symporter (BUNIF_04651) through a similar mechanism recently identified in the *B. uniformis* NP1.^50^

Since putative AP-PUL showed about 100% synteny with *P*-PUL of the *B. plebeius*, we checked possibility of horizontal gene transfer (HGT) event and found a similar genomic architecture of the PUL and its flanking regions in both the species. We found a contiguous set of mobilization and integration genes at one end of the PUL, suggesting that the PUL may be contained inside an integrative conjugative element (ICE). Subsequently, we identified a pair of identical 15-bp repeats, one of which overlapped the 3ʹ end of tRNA-lys, as it was seen in the case of *B. plebeius* (Figure 2, and supplementary excel sheet 1). However, we found a difference in the size of the ICE between *B. uniformis* JCM 13288 ^T^ (172,431 bp) and *B. plebeius* (107,705 bp). Interestingly, the *B. plebeius* ICE lacked a region present in the *B. uniformis* ICE, which contained another PUL – PUL45.

With regards to laminarin PUL in the *B. uniformis* JCM 13288 ^T^, the PUL was predicted that it can cleave linear *β-*1-3 (PUL36) and branch chain of *β-*1-6 (PUL21) linkages (Figure 2). The PUL36 consisted of surface glycan-binding proteins (SGBPs), TonB-dependent transporters (TBDT, SusC homologs), hybrid two-component system (HTCS) proteins and four enzymes (enolase, *Bu*GH3, *Bu*GH16, and *Bu*GH158), representing a typical analogous system to the starch utilization system.^51^ We further compared it with the *B. uniformis* JCM 5828 strain, which highlighted that except the newly identified GH family enzyme (*Bu*GH158), all other proteins showed 100% sequence similarity (Supplementary Figures 24). The *Bu*GH158 had amino acid level differences between the strains (Supplementary Figure 2b), suggesting sequence divergence and hence altered subtle enzymatic activities. The *Bu*GH158 in both strains was predicted to be present at the extracellular membrane by type II signal peptide prediction (SP-II, SignalP 5.0 server), although PSORTb failed to predict it. The *Bu*GH3 and *Bu*GH16 in both strains were shown to have SP-II and were predicted at the periplasmic space (PSORTb Score 9.4) and extracellular (PSORTb Score 9.7) respectively. Enolase is a part of the glycolytic pathway and predicted to occur in the cytoplasmic space (PSORTb Score 9.9). The PUL21 of *B. uniformis* JCM 13288 ^T^ has a HTCS, SusC, SusD, GH30_3 and SGBP, and is identical to the same PUL present in *B. uniformis* JCM 5828 based on JGI-IMG blast. The SusD, *Bu*GH30_3 and SGBP were predicted to be present at the extracellular membrane by PSORTb (Score ~ 9) with SP-II. The SusC has SP-I by PSORTb (Score ~9.5) for extracellular membrane, and HTCS is predicted to be present at the inner membrane, which has a SP-II tail (Figure 2).

Assuming the importance of three GHs (GH3, GH16 and GH158) associated with PUL36 and one GH (GH30_3) of PUL21 for degrading laminarin, these enzymes were cloned and heterologous expressed in *E. coli*. While we were working on these enzymes, the study of Dejean et al. (2020)^52^ appeared in which they characterized *Bu*GH3, *Bu*GH16, and *Bu*GH158. Therefore in the study, we mainly focused to work with *Bu*GH158, which is not identical to one that characterized (Supplementary Fig. S2A, B and C), and GH30_3 (Supplementary Fig. S3A and B), which was not yet characterized. Nevertheless, preliminary enzymatic assays were done with *Bu*GH3 and *Bu*GH16 as they are showing 100% similar to ones characterized from *B. uniformis* JCM 5828 (Supplementary Figures 4 and 5).

### Characterization of enzymes

**Figure 3.**
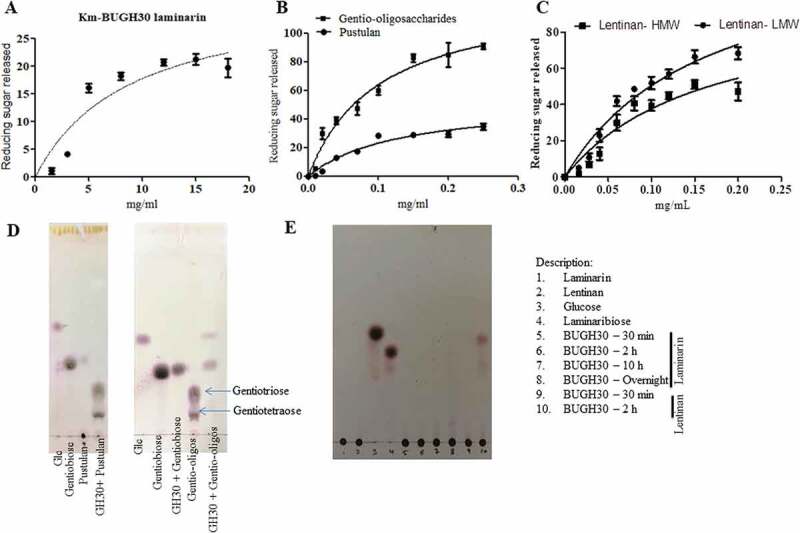
Hydrolysis of different *β-* glucans and Michaelis-Menten kinetics of *Bu*GH30_3. (a) Michaelis-Menten plot demonstrating the enzyme kinetic of laminarin. (b) Michaelis-Menten plot demonstrating the enzyme kinetics of gentio-oligosaccharides and pustulan. (c) Michaelis-Menten plot demonstrating the enzyme kinetics of lentinan LMW and HMW. (d and e) Thin layer chromatography analysis was carried out on Silica Gel 60 F254 (Merck) and generated mono- and oligo-saccharides from different substrates were visualized by spraying TLC with 5% H_2_SO_4_ in ethanol, followed by charring. Kinetics curves were analyzed on GraphPad Prism software. Three independent replicates were used for Michaelis-Menten kinetics. Bar represent average value of three independent replicates with standard errors.

The *Bu*GH30_3 is an outer surface located enzyme and was initially screened against laminarin as it was thought that it may be involved in the cleavage of *β-*1,6 linked glucose. However, it showed very weak activity on laminarin with K_m_ value of 8.863 mg/ml and with a K_cat_/K_m_ of about 5.4 (Table 1 and Figure 3(a)). Thus, further investigation on sequence of GH30_3 was made through NCBI-BLASTP programs and Phyre2 orthologous analyses, which showed 72% sequence identity with a protein (PDB- 5NGK) characterized from the *B. thetaiotaomicron* VPI-5482. Tertiary structure homology model of *Bu*GH30_3 with 5NGK suggested that there are significant changes in the surface topology of the structures of both enzymes with subtle change in the direction of side chain of Glu231 residue, corresponding to Glu138 residue in 5NGK (Supplementary Fig. S3B). Typically, catalytic domain adopted by TIM β barrel with conserved catalytic amino acid residues suggested that it belonged to the GH-A clan. The *Bu*GH30_3 is predicted to form an U-shaped substrate binding site (Supplementary Fig. S3B) that is very suitable for binding of U-shaped *β-*1,6 linked fungal glucans rather than spiral chain of *β-*1,3 linked glucans.^53^ This indicates that this enzyme may catalyze longer chain β-1,6 linked glucan as substrate. Thus, we screened the *Bu*GH30_3 with pustulan and generated a range of oligosaccharides during 1 h incubation (Figure 3(d)). These oligosaccharides were removed from undigested polysaccharide fraction by centrifugation and again used for enzymatic assay to determine product limit digestion capability (Figure 3(a and d)). Observed limit digestion products were gentiobiose and glucose (Figure 3(d)). The *Bu*GH30_3 showed 2.4-fold higher activity on oligosaccharides than on high molecular weight (HMW) polysaccharides. Two different fractions were obtained by centrifugation of purchased lentinan, supernatant and pellet fractions were called as low molecular weight (LMW) and HMW, respectively. Enzymatic activity was 1.5-fold higher with LMW as compared to HMW fractions (Table 1 and Figure 3(c)). HMW polysaccharides are less soluble in water and could provide hindrance on binding of enzyme to substrate, thus lowering the catalytic activity. These results indicate that the enzyme acts on fungal *β-*1,6 linked glucans rather than on macroalgal and plant-derived glucans having *β-*1,3 linkage with single *β-*1,6 linked residue. Activity of the *Bu*GH30_3 on lentinan further provides evidence toward structure of lentinan, highlighting that lentinan must have long chain of *β-*1,6 linked glucose residues as also recently characterized.^16^ It suggests that identifying such enzymes is also essential for chemical characterization of carbohydrates.

**Table 1.** Kinetic parameters for the hydrolysis of various substrates by GH158 and GH30.

Enzyme	Substrate	K_m_ (mg mL^−1^)	K_cat_ (s^−1^)	K_cat_/K_m_ (s^−1^ mg^−1^ mL)
*BU*GH158	Laminarin	0.27 ± 0.14	40.83 ± 2	151.2
	Curdlan- processed-A	0.29 ± 0.07	23.39 ± 3	80.66
	Curdlan- processed-B	0.74 ± 0.25	16.08 ± 0.8	21.72
	Curdlan- native	0.87 ± 0.3	9.7 ± 0.5	11.15
	Yeast β-glucan	0.95 ± 0.41	7.7 ± 0.5	8.1
	Lichenin	0.6 ± 0.23	26.87 ± 1.6	45
*BU*GH30	Gentio-oligosaccharides	0.1 ± 0.02	183.2 ± 4.5	1830
	Pustulan (HMW)	0.13 ± 0.03	75.6 ± 2.3	582
	Laminarin	8.863 ± 3.4	47.86 ± 2.2	5.4
	Lentinan (LMW)	0.1632 ± 0.04	187.8 ± 4.8	1160
	Lentinan (HMW)	0.1627 ± 0.06	142.0 ± 5.6	873

**Figure 4.**
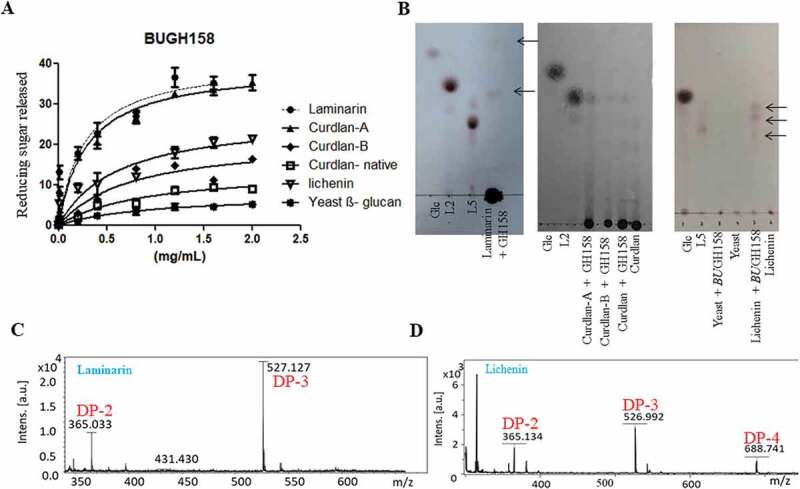
(a) Hydrolysis of different *β-* glucans and Michaelis-Menten kinetics of *Bu*GH158. (b) Thin layer chromatography analysis was carried out on Silica Get 60 F254 (Merck) and generated mono- and oligo-saccharides were visualized by spraying TLC with 5% H_2_SO_4_ in ethanol, followed by charring. Kinetics curves were analyzed on GraphPad Prism. MALDI-TOF mass spectrum was performed in positive ion mode of enzymatic products from laminarin (c) and lichenin (d). All masses were observed with sodium attached molecular mass [M+ Na]^+^. Three independent replicates were used for Michaelis-Menten kinetics. Bar represent average value of three independent replicates with standard errors.

The *Bu*GH158 was predicted to cleave 1–3 linkage of curdlan. It showed very weak activity against native curdlan; therefore, it was pretreated with two different methods. With the pre-treatment, K_m_ of curdlan-B was slightly reduced, while K_m_ of curdlan-A was dropped to approximately threefold (Figure 4(a)). As a result of pre-treatment, K_cat_ of curdlan-A and curdlan-B increased around 2.5 to 1.7-fold, respectively. Since curdlan is less soluble in water and in order to rationally check impact of the solubility problem, the *Bu*GH158 was tested with laminarin and it showed same magnitude of K_m,_ but K_cat_ was increased by twofold. These patterns highlight that enzyme could not properly access the binding site on the substrate when curdlan is present in triple helix conformation (Figure 4). During the digestion of laminarin, it can generate DP-1 to DP-3 oligosaccharides from laminarin as final product, whereas it generated a variety of oligosaccharides from curdlan-A (Figure 4(b and c)). Successively, Michaelis-Menten kinetics of *Bu*GH158 with yeast *β-* glucan and lichenin at the optimum pH and temperature (Supplementary Fig. S6) revealed that K_cat_/K_m_ is about 6-fold magnitude higher for lichenin than yeast *β-* glucan. Three different major oligosaccharides were detected from lichenin, i.e. DP-2 to DP-4 (Figure 4(b and d)). These assays confirmed that GH158 is an endolytic enzyme with broad spectrum hydrolytic potential.

Sequence alignment and secondary structure information revealed that there are several amino acids differences in the *Bu*GH158 of the *B. uniformis* JCM 13288 ^T^ from the GH158 of the *B. uniformis* JCM 5828. Therefore, tertiary structure homology modeling of *Bu*GH158 was performed with PDB-6PAL using SWISS-MODEL, which further confirmed that there are some differences in surface topology of structures of both enzymes but had the same conserved catalytic site. Especially, *Bu*GH158 of the *B. uniformis* JCM 13288 ^T^ has identical position of residues that involved in acid/base exchange (E118 corresponding to E137 of 6 PAL) and nucleophile attack (E201 corresponding to E220 of 6 PAL) during a canonical Koshland double displacement mechanism (Supplementary Fig. S2C).

The *Bu*GH16 showed activity on laminarin, lentinan, and curdlan (Supplementary Fig. S7). The enzymatic activity of GH16 was observed up to 2 h and detected limit-digest products were laminaribiose, laminaritriose, and laminaritetroase from laminaripentaose, while laminaritriose and laminaritetroase were obtained from digestion of curdlan (Supplementary Fig. S7). Additionally, DP-2, DP-3, and DP-4 oligosaccharides from laminarin digestion and DP-2, and DP-3 from lentinan digestion were observed when analyzed through ESI-MS (data not shown). Interestingly, Phyre2 analysis modeled the *Bu*GH3 based on an enzyme (PDB-3U48), which was extracted from soil compost and acted on *β-*1,4 linked oligosaccharides. Contrarily, it made distinct clade in phylogenetic analysis (Supplementary Fig. S4A). The *Bu*GH3 of *B. uniformis* JCM 13288 ^T^ showed high activity on *β-*1,3 linked laminaribiose as compared to *β-*1,6 linked gentiobiose and *β-*1,4 linked cellobiose (Supplementary Fig. S8). Enzymatic activity, and alignment of amino acid sequences suggested that *Bu*GH16 and *Bu*GH3 of both, *B. uniformis* JCM 13288 ^T^ and *B. uniformis* JCM 5828, were very similar to each other, and phylogenetic analysis showed some degrees of similarity with GHs present in marine or environmental associated bacteria (Supplementary Fig. S4A and S5A).

### Production and characterization of β-1, 3-OSs from curdlan

**Figure 5.**
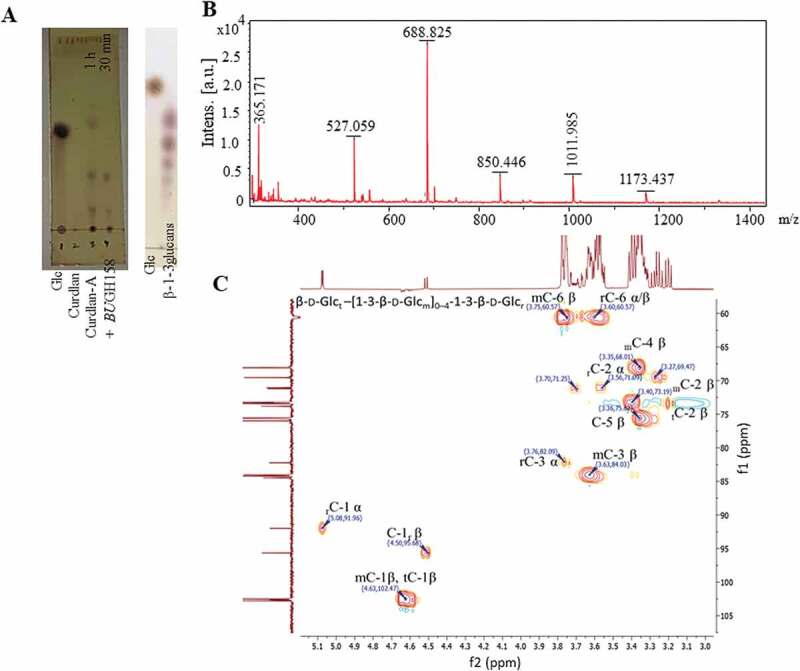
Generation of different *β-*1, 3- oligosaccharides (*β-*1, 3-OSs) and characterization. (a) Generation of *β-*1, 3-OSs and final product after purification on thin layer chromatography. (b) MALDI-TOF mass spectrum analysis of purified *β-*1, 3-OSs. All masses were observed with sodium attached molecular mass [M+ Na]^+^. (c) ^1^H-^13^C heteronuclear single quantum coherence spectroscopy of purified *β-*1, 3-OSs.

End products of curdlan digestion by *Bu*GH158 depended on the time of incubation and ratios of substrate to enzyme. For generation of oligosaccharides, different ratios of curdlan-A and the *Bu*GH158 were used and we observed that lower ratios of curdlan-A to enzyme produced monosaccharide and disaccharides whereas higher ratios of curdlan-A to enzyme produced *β-*1, 3-OSs (data not shown). Therefore, 2:1 and 3:1 (curdlan-A: *Bu*GH158) were used for *β-*1, 3-OSs production where different reaction mixtures were incubated for variable periods of time ranging from 30 min to 2 h (Figure 5(a)). All the reaction mixtures were combined at the end of incubation time and enzyme was heat inactivation after incubation time and undigested residues were removed by centrifugation. After purification by column and about 310 mg of *β-*1, 3-OSs from 1 g curdlan recovered. Fractions containing DP-2 to DP-7 were collected (Figure 5(b)) and used for growing bacteria.

1D (^1^H and ^13^C-DEPT135) and 2D (COSY and HSQC) NMR confirmed presence of the *β-*1,3 linkage and glucose residues. Signals of carbon 3 in the HSQC and ^13^C-DEPT135 pinpointed the *β-* 1,3-linkage of middle (m) and reducing end (r) residues at about 84.0 ppm (Figure 5(c) and supplementary Fig. S9A and B). Anomeric signals of C1/H1 of the r, m, and terminal (t) residues of β forms in the HSQC and COSY were observed at 95.68/4.5 and about 102/4.6 ppm, respectively. Signals of non-linked carbons, C5, 2 and 4 of β forms of m residues were clearly observed at 75.65, 73.19, and 68.01, respectively. While carbon 6 of all residues was observed at about 60 ppm as inverted signals in ^13^C-DEPT135 (supplementary Fig S9A). Since anomeric C1/H1 of r-residue is in free form, it can be also present in α conformation. Thus, signals of C1/H1, C2/H2 and C3/H3 in α form of the r-residues were distinctly observed at about 91.96/5.08,71.09/3.56 and 82.09/3.76, respectively. These signals further confirmed the presence of *β-*1, 3-OSs.

### Evaluating glycan cooperation between different human gut bacteria

**Figure 6.**
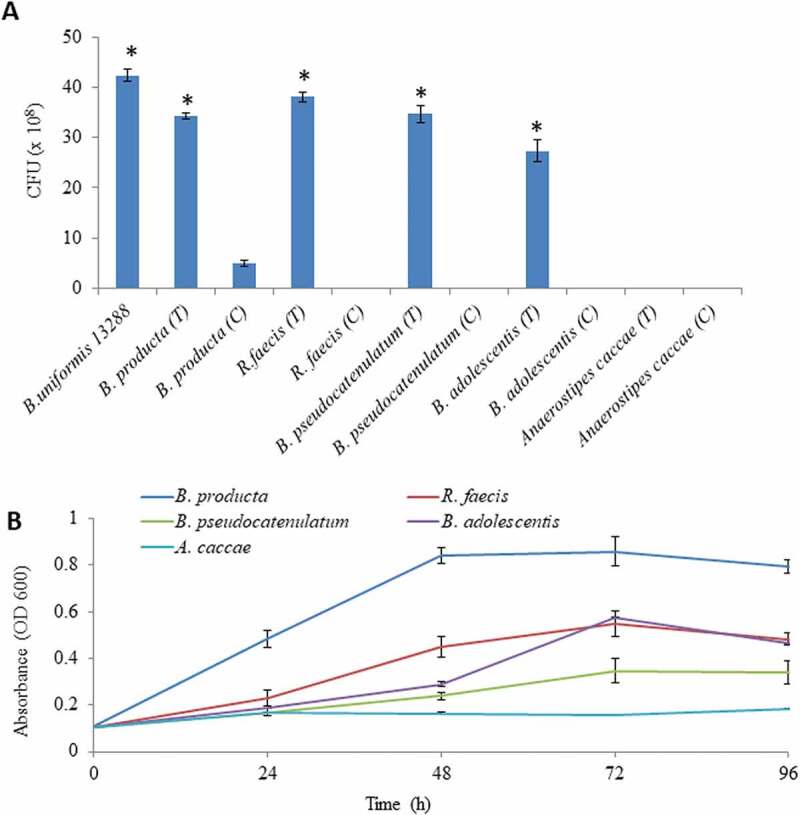
Evaluation of cooperative benefits of polysaccharide digestion by outer surface attached enzymes. (a) Growth of different bacteria in mono and co-culture on agar Petri-plates, and (b) Growth of different bacteria on purified *β-*1, 3- oligosaccharides generated by *Bu*GH158. The *Blautia producta* JCM 1471 ^T^, *Ruminococcus faecis* JCM 15917 ^T^, *Bifidobacterium pseudocatenulatum* JCM 1200 ^T^, *Bifidobacterium adolescentis* JCM 1275 ^T^ and *Anaerostipes caccae* JCM 13470 ^T^ were used. Three independent replicates were used for each experiments. Results of mono and co-culture analyses were statistically analyzed through t-test (significance level at *p* < .05).

Enzymatic patterns of *Bu*GH16 (Supplementary Fig. S7) and *Bu*GH158 (Figure 4(b-d)) indicated that both enzymes can produce oligosaccharides, which can be utilized by other bacteria present in their vicinity.^52^ Especially, such cooperativity is rarely seen with Gram-positive bacteria. In order to check this hypothesis, Gram-positive bacteria were spotted near to *B. uniformis* JCM 13288 ^T^ on agar plate and subsequently, CFUs were calculated. After 5 days of incubation, CFUs of *B. producta* JCM 1471 ^T^, *R. faecis* JCM 15917 ^T^, *B. pseudocatenulatum* JCM 1200 ^T^ and *B. adolescentis* JCM 1275 ^T^ were significantly increased whereas *A. caccae* JCM 13470 ^T^ did not grow (Figure 6(a)). Bacterial growth patterns were congruent with the profile on generated DP-2 to DP-7 (Figure 6(b)).

Surprisingly, *B. producta* JCM 1471 ^T^ showed strong growth on *β-*1, 3-OSs in co-culture condition (Figure 6(a)). Although, it did not independently grow well on laminarin. Genomic evidence suggests that it has homologous genes to *Bu*GH3, *Bu*GH30_3 and *Bu*GH16 (Supplementary Table S2). In addition, it has a gene coding for putative laminarin phosphorylase. The signalP-5.0 predicted that only *BP*GH16 is present at the outer membrane that is likely to convert polysaccharide in to oligosaccharides before its import into the cytoplasm. In order to check possibility to cleave *β-*1, 6 linked glucan, we cloned a best heat gene (JGI-IMG – protein ID- 2515950412, homologous to *Bu*GH30_3) but it did not cleave any tested substrates (Supplementary Fig. S10), suggesting that it might be not cleaving branch chain of *β-*1, 6 linked glucan. To the best of our knowledge, this is the first evidence to suggest that human gut Gram-positive bacteria can have *β-*1, 3 linked glucan digesting gene cluster.

## Discussion

Macroalgae is commonly used as dietary fibers in Japanese population every day with an estimate of 14.2 g per person per day – mainly in the form of nori and wasabi.^54^ These dietary fibers are mainly utilized by the human gut bacteria, in which *Bacteroides* is predominantly present^1^ and renders unprecedented benefits for gut health.^2,7^ The array of CAZymes present in *Bacteroides* is highly diverse^7^ and there is a growing understanding that dietary fibers act as a selective pressure to acquire unique genes or gene clusters from other environments. In order to further reinforce this concept, we sequenced a genome of the Japanese gut bacterium, *B. uniformis* JCM 13288 ^T^, which is shown to be unique in a sense that it can robustly grow on macroalgal glycans such as laminarin, agarose, porphyran (Figure 2(e)), and fungal derived glucan (Supplementary Fig. S11). Its genome sequence was compared with *B. uniformis* JCM 5828, wherein AP-PUL was found to be uniquely present only in the JCM 13288 ^T^ strain that enabled it to grow on porphyran and agarose. Astonishingly, AP-PUL of the *B. uniformis* JCM 13288 ^T^ showed 100% synteny to the *P*- PUL present in *B. plebeius*. We looked for homology of some of the genes of AP-PUL in public domain (NCBI-BlastP), which matched mostly with marine bacteria with varied degree of identities, 24 to 60% (Supplementary excel sheet 1). This observation was congruent with previous findings, which suggest that enrichment of CAZymes for utilization of macroalgae in *Bacteroides* most likely happened through HGT-ICE events from marine bacteria that are commonly consumed along with food/dietary fibers.^55,56^ PULs for agarose/porphyran digestion are highly abundant in the metagenomes of Japanese populations;^55^ however, the ICE containing the AP-PUL identified in our study is unique and contains an additional PUL (PUL45) (Figure 2(c) and supplementary excel sheet 1). We hypothesize that the common regions contained within the ICEs may be the ancestral architecture of the ICE and the *B. uniformis* JCM13288^T^ strain contains a second ICE (containing PUL45) that integrated within the ancestral ICE without disrupting the functionality of PUL46 that allowed the strain to grow on porphyran. The PUL45, based on genomic analysis, is predicted to be involved in utilization of chondritic sulfate/heparin sulfate and a gene (BUNIF_04562) near to the 3ʹ end of tRNA-lys, which showed 40% homology to *β*- agarase (PDB-5T3B) of the *B. plebeius*, present in another location of the genome. Further studies may be required to check the activity of this identified β- agarase gene. Therefore, the genomic architecture of this ICE is unique and to the best of our knowledge has not been reported from any other *Bacteroides* species. Further functional genomics investigations may throw light on the additional role(s) of this ICE region apart from agarose/porphyran utilization.

**Figure 7.**
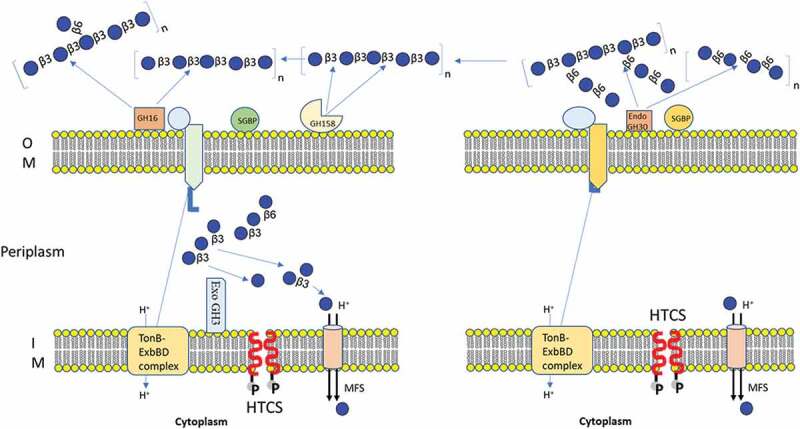
Schematic model of the laminarin and pustulan utilization pathways. Prediction of cellular location of proteins were based on TMHMM Server, v. 2.0 and SignalP, v. 4.1.^42^ (a) Initial degradation of laminarin takes place by two outer membrane anchored proteins (*Bu*GH158 and *Bu*GH16), which transform polysaccharides into *β-*1, 3-OSs before importing them into the periplasmic space. The SGBP is involved in capturing of *β-*1, 3-OSs, which were generated after cleaved by BuGH158 or BuGH16 from curdlan, laminarin, lentinan or lichenin. SGBP provides access of these oligosaccharides to SusCD complex, which eventually imports *β-*1, 3-OSs into periplasmic space. A *Bu*GH3 is present in the periplasm that converts *β-*1, 3-OSs in to monomers before sending them to the cytoplasm via the Major Facilitator Superfamily (MFS) transporter. (b) Initial degradation of pustulan takes place by *Bu*GH30_3 that cleaves *β-*1, 6- linked polysaccharides (pustulan and lentinan) into *β-*1, 6-OSs with the help of SGBP (65% homologous to *Bacteroides thetaiotaomicron* VPI-5482) and then imports them into the periplasmic space via the SusCD complex. Latter locus lacks exo-acting enzyme, therefore, we hypothesized that it may utilize via an enzyme (*Bu*GH3) expressed in laminarin- PUL.

In this study, we characterized enzymes associated with laminarin and pustulan PULs. Laminarin- PUL encodes two outer membrane-anchored proteins (*Bu*GH158 and *Bu*GH16), which transform polysaccharides into *β-*1, 3-OSs before importing it into the periplasmic space. The SGBP is involved in capturing of *β-*1, 3-OSs, which are generated enzymatically after hydrolyzing curdlan, laminarin, lentinan or lichenin, before providing access to SusCs. A *Bu*GH3 present in the periplasm converts *β-*1, 3-OSs into monomers before sending to the cytoplasm via the Major Facilitator Superfamily (MFS) transports (Figure 7). Secondary structural alignment similarity and homology modeling based on Phyre2 of *Bu*GH16 suggested that it can tolerate larger branched chain β-1,3 and β-1,6 glucan (such as lentinan) in gut environment, leading to generation of oligosaccharides (Supplementary Fig. S7). Capability of adopting long branched chain glucan by *Bu*GH16 was also convincingly demonstrated by Dejean et al. (2020).^52^ The *Bu*GH158 has for the first time been used for generation of *β-*1, 3-OSs after optimization of the type of curdlan. Pustulan-PUL in the *B. uniformis* JCM 13288 ^T^ encodes single *Bu*GH30_3 that cleaves *β-*1, 6-linked polysaccharides (pustulan and lentinan) in to *β-*1, 6-OSs with the help of SGBP (65% homologous to *B. thetaiotaomicron* VPI-5482) and then imports it into the periplasmic via SusCD complex (Figure 7). The latter locus lacks exo-acting enzyme, therefore, we hypothesized that it may utilize via an enzyme (*Bu*GH3) expressed in laminarin- PUL. Though, it has very weak activity with gentiobiose generated in periplasm from laminarin either by debranching or limit product of *β-*1, 6-linked glucans. It seems to be an authentic gene coding *Bu*GH3 that is responsible for cleaving gentiobiose. The above observation also indicates that a higher concentration of imported *β-*1, 6-OSs may be required to activate non-cognate HTCS. Notwithstanding, we searched for all GH3 in the *B. uniformis* JCM 13288 ^T^ and annotated 33 of them. Out of them, 3 genes (BUNIF_00608-PUL5, BUNIF_00462-PUL1 and BUNIF_04929) were shown to have 74, 58, and 58% homology to BT3314, respectively. BT3314 (GH3) was previously identified as a part of fungal *β-*1,6 utilization locus in the *B. thetaiotaomicron* VPI-5482.^37^ Protein localization predicted that all the three proteins were present in the periplasmic space, therefore, it can be assumed that at least one of them might be involved in utilization of *β-*1, 6-OSs, produced by *Bu*GH30_3 present in the PUL21 (Figure 7). However, this has not yet been experimentally established.

Laminarin – PUL was observed to be involved in the cross-feeding system with Gram-positive bacteria, confirming that all generated *β-*1, 3-OSs by *Bu*GH158 or *Bu*GH16 are not imported into periplasmic space but can act as public goods to promote other gut bacteria. In order to seek genomic evidence for utilizing *β-*1, 3-OSs by used strains in the study, we did NCBI-BlastP search against genomes of them through JGI-IMG. *Bu*GH3 had shown highest homology with some genes of these strains, indicating that those can cleave either β-1-3 or β-1-6 linkages. For example, *B. adolescentis* JCM 1275 ^T^ previously shown to grow on curdlan and laminarin,^57^ and *R. faecis* JCM 15917 ^T^ is predicted to cleave β-1-3 linkage either by putative β- glucosidase or laminarin phosphorylase (Supplementary Table S2). A gene of *B. pseudocatenulatum* JCM 1200 ^T^ showed homology (82%) with a protein obtained from *Bifidobacteria longum* that can cleave β-1-6 linkage,^58^ postulating that the gene is likely to act on gentiobiose. This might be a reason for slow growth pattern on β-1, 3-OSs (Figure 6(b)). Interestingly, *B. producta* JCM 1471 ^T^ was discovered as a potential strain, which was found to hydrolyze *β-*1, 3-OSs. Certain *B. producta* strains produce lantibiotic that can inhibit colonization by Gram-positive pathogenic bacteria such as vancomycin-resistant *Enterococcus faecium*.^59^ Identifying the capability of *β*-1, 3-OSs utilization by such species may further strengthen their use as potential probiotics.

Laminarin- PULs showed same genetic synteny in both *B. uniformis* strains and genetic variability with other *Bacteroides* (Supplementary Fig. S13 and supplementary excel sheet 1). Interestingly, *B. fluxus* lacks GH16 and others lack GH158 but showed growth on laminarin.^52^ The pustulan-PUL is also pervasive among *Bacteroides* and other genera regardless of presence of α-mannan utilization locus.^37^ This PUL is most likely to be involved in utilization of dietary edible mushroom and utilization of cell wall glycans of gut commensal fungal species such as *Saccharomyces cerevisiae*. Adaptation of the pustulan-PUL among gut commensal bacteria is also useful for controlling the population of opportunistic pathogenic fungi such as *Candida glabrata* and *Candida albicans* as they undergo remodeling of cell wall polysaccharides.^60^

Although consumption of macroalgal glycans is popular among coastal and Japanese populations since ancient times in China and Japan, utilization of such glycans has increased globally over the centuries due to rising awareness about health implication because of their protective effects against type 2 diabetes, obesity, and cardiovascular diseases.^61^ Macroalgal glycans are distinct from other refractory plant-derived glycans^62^ and require unique genes in the human gut bacteria to thrive on them. Because of regular utilization of macroalgal glycans, human gut bacterial genomes were upgraded by some clusters of marine bacteria that subsequently engrafted into other species without changes in genetic integrity, as revealed from phylogenetic analyses of *Bu*GH3, *Bu*GH16, and *Bu*GH158 (Supplementary Fig. S2A, S3A and S4A), or genetic modification including changes in orientation, deletion, or addition of genes (Supplementary Fig. S13). Additionally, availability of variety of dietary nutrients in human diet (such as mushroom, bacterial glucans, and yeast cell wall) forces gut microbiota to acquire potential genes (such as PUL 21) from other environments. There are also chances that existing enzymes might be modified (via direct evolution or mutation) in a way that they can digest more than one glycan, such as *Bu*GH158 and *Bu*GH16 can digest diverse *β*- glucans. Also, generated *β*-1, 3-OSs by the activity of *Bu*GH158 promoted growth of several Gram-positive bacteria. Thus, the study provides evidence that *β*-1, 3- glucans utilization genes have been transferred not only into *Bacteroides* but also into Gram-positive bacteria. Diverse *β*- glucans utilization PULs identified in this study may pave the way for the development of engineered functional foods for the improvement of human health through proper nutritional intervention therapy.

## Supplementary Material

Supplemental Material
