# Supplementary material for: Linear and branched β-Glucans degrading enzymes from versatile Bacteroides uniformis JCM 13288T and their roles in cooperation with gut bacteria
Source: Gut Microbes. 2020 Oct 10;12(1):1826761. doi: 10.1080/19490976.2020.1826761 (PMC7553746; doi:10.1080/19490976.2020.1826761)
Supplement: Supplemental Material [file KGMI_A_1826761_SM4192.zip › Supplementary information/Supplementary.docx]

**Linear and branched *β*-Glucans degrading enzymes from versatile *Bacteroides uniformis* JCM 13288^T^ and their roles in cooperation with gut bacteria**

**Ravindra Pal Singh^1*^, Sivasubramanian Rajarammohan^2^, Raksha Thakur^1^ and Mohsin Hassan^1^**

^1^Food and Nutrition Biotechnology Division, National Agri-Food Biotechnology Institute, Mohali, Punjab, India

^2^Agricultural Biotechnology Division, National Agri-Food Biotechnology Institute (NABI), SAS Nagar, Punjab, 140306, India.

**^*^**For correspondence: [ravindrapal.1441@gmail.com](mailto:ravindrapal.1441@gmail.com); r.p.singh@nabi.res.in


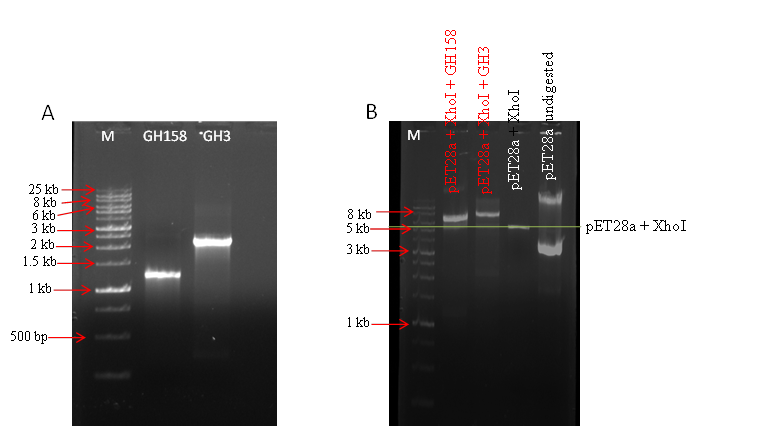


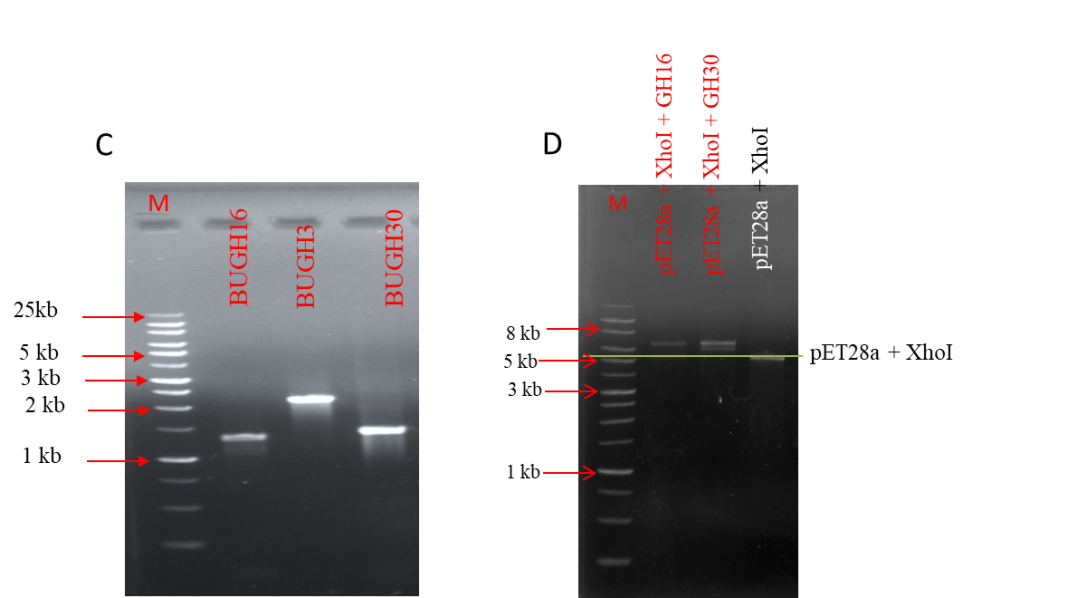


**Fig. S1**. Enzymatic genes of interest were amplification from *Bacteroides uniformis* JCM 13288 by polymerase chain reaction (PCR). Molecular size of pET28a is 5369. A and C- PCR amplified products and B and D- confirmation of inserts in to pET28a vector.

*B. uniformis* JCM 5828

*B. uniformis* JCM 13288^T^

*Victivallis vadensis*

*Bacteroides fluxus* YIT 12057

*Bacillus halodurans* (GH81)

*Lentinula edodes* (GH128)

*Kribbella flavida* (GH64)

*Cellulosimicrobium cellulans* (GH16)

*Arthrobacte*r sp. (GH55)

94

95

97

100

100

100

0.20

GH158

**Fig S2A.** Phylogenetic analysis of predicted *β*- 1-3 glucanase (*Bu*GH158) from *Bacteroides uniformis* JCM 13288^T^ with other homologous amino acid sequences of different glycoside hydrolyses. The tree was inferred using the Neighbor- Joining method[^1^](#_ENREF_1). The sum of branch length is 6.07034456 and values of bootstrap are marked next to the branches[^2^](#_ENREF_2). The evolutionary distances in the tree were computed using the Poisson correction method[^3^](#_ENREF_3), which were calculated based on units of the number of amino acid substitutions per site. All ambiguous positions were removed for each sequence pair (pairwise deletion option) before tree was processed. Evolutionary analyses were conducted in MEGA-X[^4^](#_ENREF_4). GH- glycosidase hydrolyse, B- *Bacteroides*


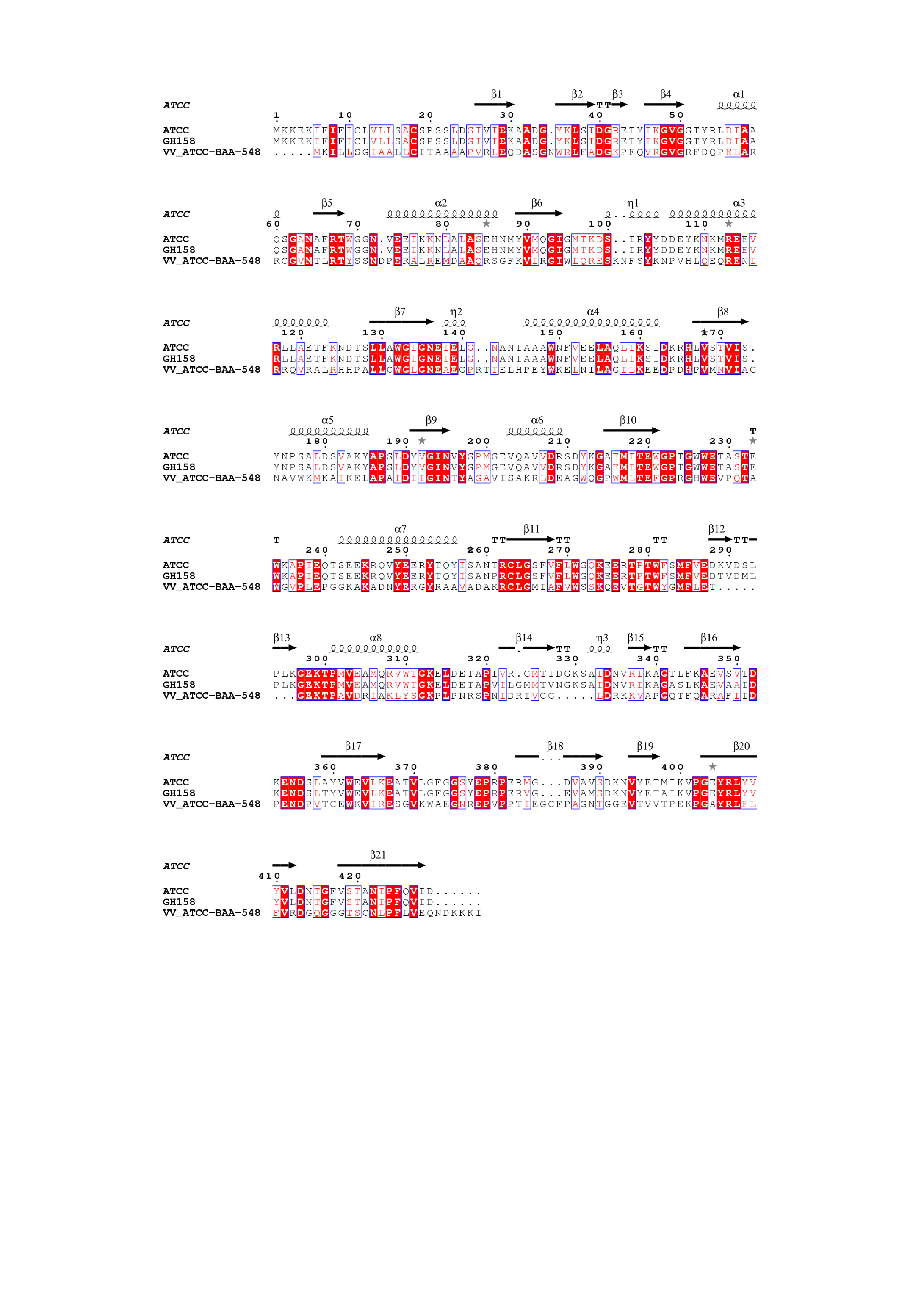


**Fig. S2B.** Structural information of the *β*- 1-3 glucanase (*Bu*GH158) from *Bacteroides uniformis* JCM 13288^T^. ClustalW and ESPript 3.0 were used for sequence alignment and secondary structure information on aligned sequences, respectively. Selection of amino acid sequences based on Phyre 2 orthologous analysis that showed 94% sequence identity was used in this figure. Amino acid sequences of *Bacteroides uniformis* JCM 5828 (ATCC 8492), and *Victivallis vadensis* ATCC BAA-548 were taken from CAZy database (<http://www.cazy.org/GH158.html>). The PDB-6PAL (GH158) of the *Bacteroides* *uniformis* was used for deducing amino acid residues in secondary structures of *Bu*GH158.


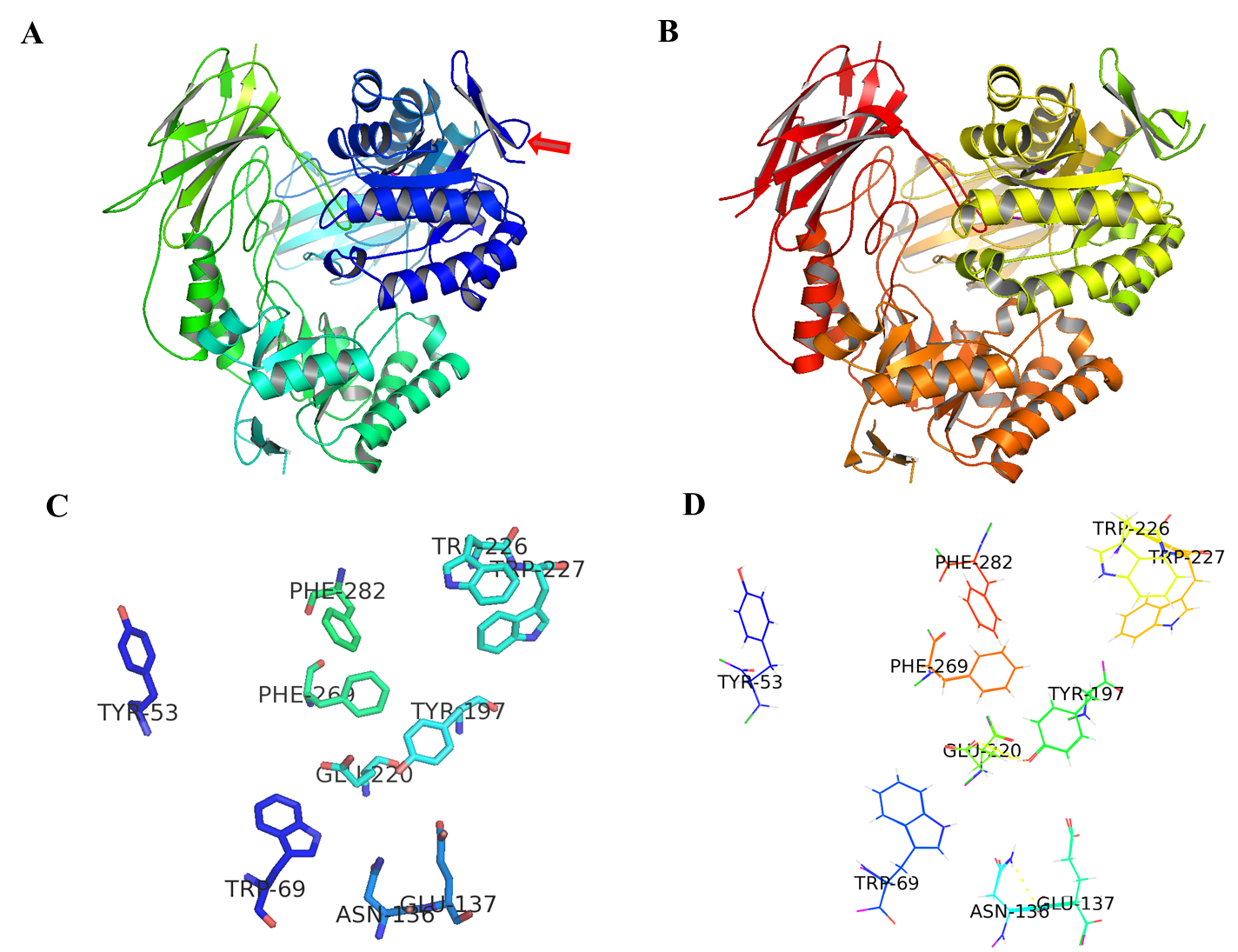


**Fig. S2C.** Tertiary structure homology modelling of *Bu*GH158 in cartoon representation. (A) Homology modelling of *Bu*GH158 of *Bacteroides uniformis* JCM 13288^T^ based on SWISS-MODEL software[^5^](#_ENREF_5). (B) Tertiary structure of PDB: 6PAL (GH158) of the *Bacteroides* *uniformis* JCM 5828 (ATCC 8492). (C) Active site of 6PAL with catalytic residues (Glu137 and Glu220) with other aromatic residues. (D) Superposition of active site of GH158 of *Bacteroides uniformis* JCM 13288^T^ and 6PAL of the *Bacteroides* *uniformis* JCM 5828. The conserved catalytic residues of both enzymes are shown as sticks through technical option in PYMOL. Surrounding aromatic amino acids residues in the active site of the GH158 was similar to that of PDB-6PAL[^6^](#_ENREF_6), suggesting that it can favor a substrate having single *β-*1,6 linked glucose residue on a linear main chain rather than a longer branch chain, such as in yeast *β-* glucan.


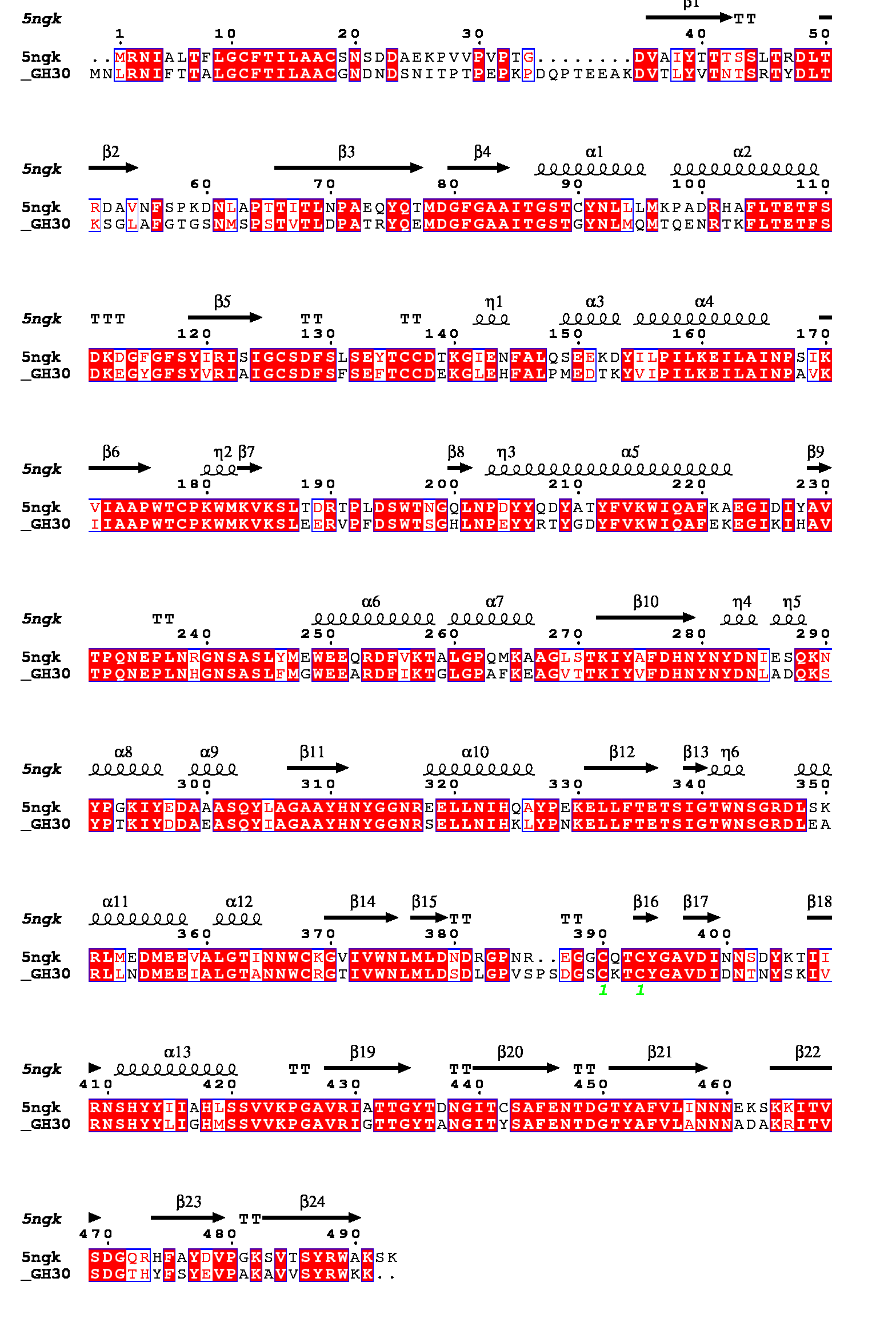


**Fig. S3A.** Structural information of the endo *β*- 1-6 glucanase (*Bu*GH30_3) from *Bacteroides uniformis* JCM 13288^T^. ClustalW and ESPript 3.0 were used for sequence alignment and secondary structure information on aligned sequences, respectively. An amino acid sequence (PDB: 5NGK) of *B*. *thetaiotaomicron* based on Phyre 2 orthologous analysis that showed 72% sequence identity was used in this figure for deducing amino acid residues in secondary structures of *Bu*GH30_3. Amino acid sequence of GH30_3 from *Bacteroides uniformis* JCM 13288^T^, and *B*. *thetaiotaomicron* were taken from genome sequence and NCBI respectively.


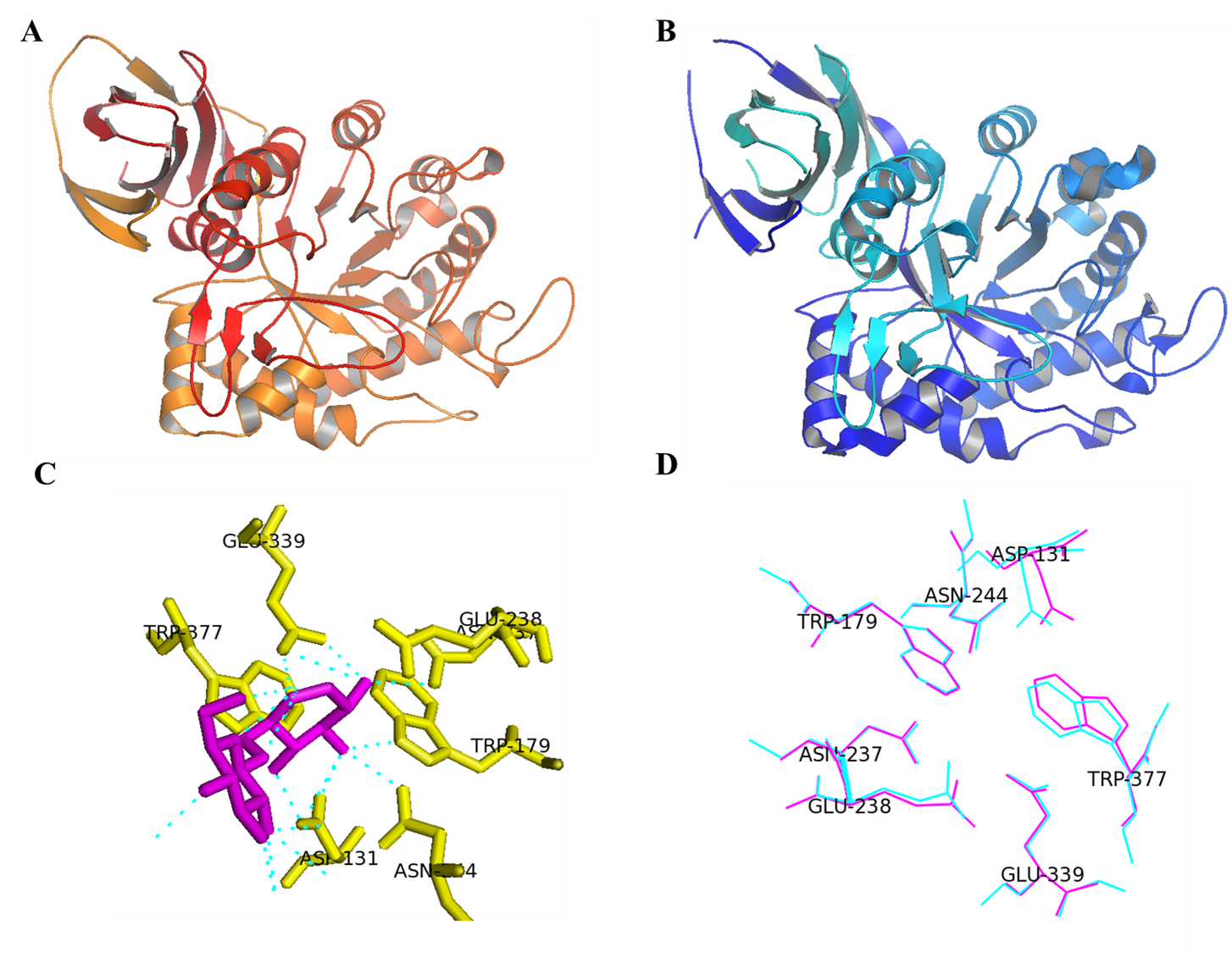


**Fig. S3B.** Tertiary structure homology modelling of *Bu*GH30_3 in cartoon representation. (A) Homology modelling of *Bu*GH30_3 based on SWISS-MODEL software[^5^](#_ENREF_5). (B) Tertiary structure of PDB: 5NGK (GH30) of *Bacteroides* *thetaiotaomicron* VPI-5482. (C) Active site of 5NGL of *B.* *thetaiotaomicron* with ligand *β*-glucosyl-1, 6-deoxynojirimycin, a weak inhibitor of enzyme[^7^](#_ENREF_7). (D) Superposition of residues of active site of BuGH30_3 of *Bacteroides uniformis* JCM 13288^T^ (cyan) and 5NGK (GH30) of *B. thetaiotaomicron* VPI-5482 (magenta). The conserved catalytic residues of both enzymes are shown as sticks through technical option in PYMOL.

*B. uniformis* JCM 5828 (GH3)

*B. uniformis* JCM 13288^T^

*B. uniformis* An67 (GH3)

*Listeria innocua* Clip11262 (GH3)

Metagenome- (PDB-3U48), GH3

*B. intestinalis* (PDB- 5TF0), GH3

*Pseudoalteromonas* sp. BB1 (GH3)

*Aspergillus oryzae* RIB40 (GH5)

*Streptomyces* sp. Sirex AA-E (GH55)

100

100

55

100

100

56

0.20

**Fig S4A.** Phylogenetic analysis of predicted *β*- 1-3 glucosidase (*Bu*GH3) from *Bacteroides uniformis* JCM 13288^T^ with other orthologous amino acid sequences of different glycoside hydrolyses. The tree was inferred using the Neighbor-Joining method[^1^](#_ENREF_1). The sum of branch length is 4.91649693, and values of bootstrap are marked next to the branches^2^. The evolutionary distances in the tree were computed using the Poisson correction method[^3^](#_ENREF_3), which were calculated based on units of the number of amino acid substitutions per site. All ambiguous positions were removed for each sequence pair (pairwise deletion option) before tree was processed. Evolutionary analyses were conducted in MEGA-X ^[4](#_ENREF_4)^. GH- glycosidase hydrolyse, B- *Bacteroides*


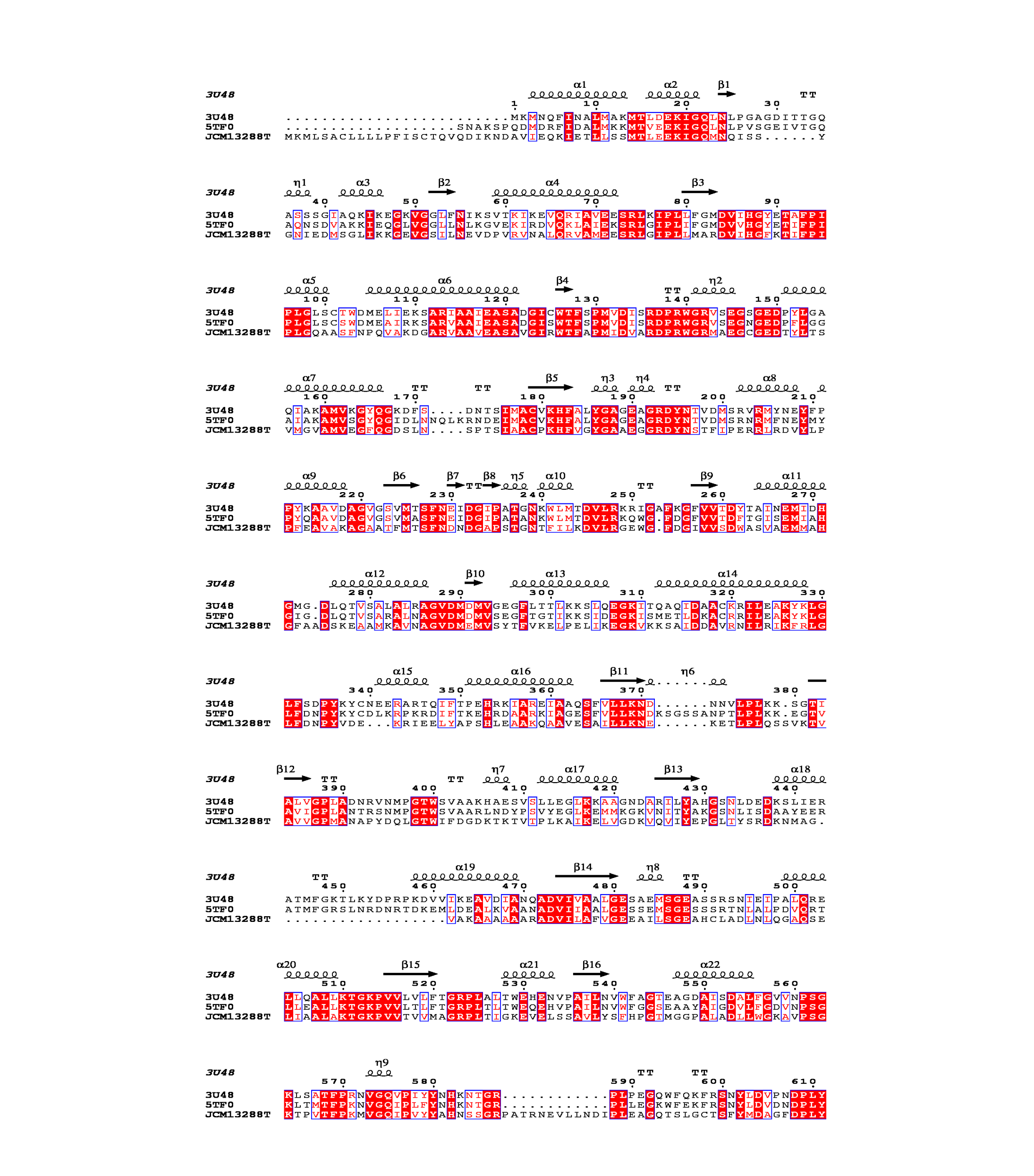


**
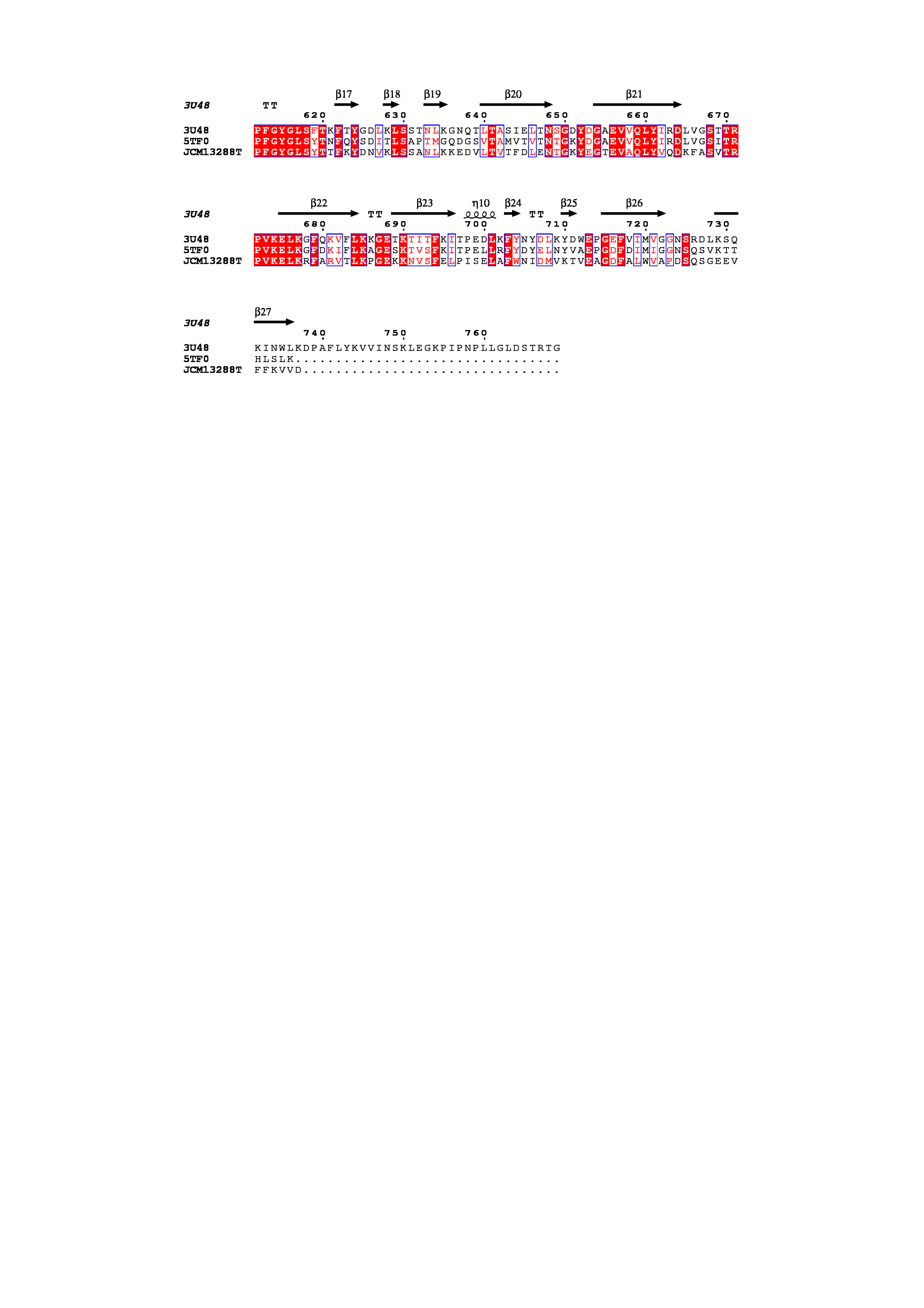
**

**Fig. S4B.** Structural information of the *β*- 1-3 glucoside (BuGH3) from *Bacteroides uniformis* JCM 13288^T^. ClustalW and ESPript 3.0 were used for sequence alignment and secondary structure information on aligned sequences, respectively. Two sequences, based on Phyre 2 homology analysis that showed more than 40% sequence identity, were used in this figure. Amino acid sequences (taken from UniProt software) of PDB-3U48 and 5TF0 were belonged to soil compost metagenomic sample and *Bacteroides intestinalis* respectively*.* The PDB-3U48 was used for deducing amino acid residues in secondary structures of *Bu*GH3.

*Pyrococcus furiosus*

*Thermotoga maritima* ATCC 43589

*Nocardiopsis* sp. F96

*Rhodothermus marinus*

*Zobellia galactanivorans* DSM 12802

*B. uniformis* JCM 13288^T^

*B. uniformis* JCM 5828

*B. ovatus* ATCC 8483

*B. uniformis* JCM 13288^T^ (*Bu*GH158)

100

99

97

83

66

47

0.20

**Fig. S5A.** Phylogenetic analysis of predicted *β*- 1-3 glucanase (BuGH16) from *Bacteroides uniformis* JCM 13288^T^ with other orthologues amino acid sequences of GH16 recovered from other strains. These sequences showed more than 30% sequence identity based on Phyre 2 homology analysis. As outgroup *Bu*GH158 was used. The tree was inferred using the Neighbor-Joining method[^1^](#_ENREF_1). The sum of branch length is 5.05141816, and values of bootstrap are marked next to the branches[^2^](#_ENREF_2). The evolutionary distances in the tree were computed using the Poisson correction method[^3^](#_ENREF_3), which were calculated based on units of the number of amino acid substitutions per site. All ambiguous positions were removed for each sequence pair (pairwise deletion option) before tree was processed. Evolutionary analyses were conducted in MEGA-X[^4^](#_ENREF_4). GH- glycosidase hydrolyse.


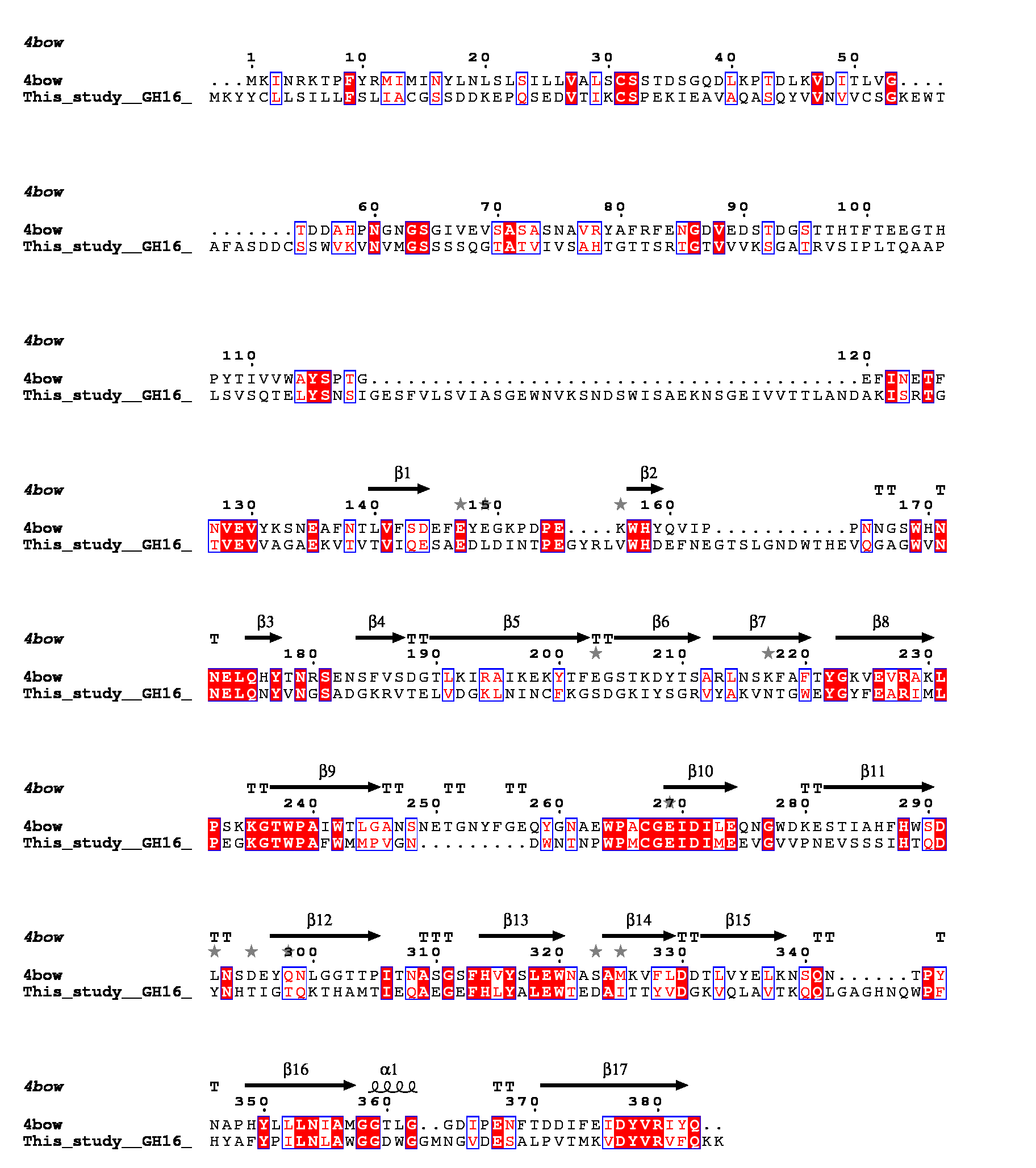


**Fig. S5B.** Structural information of the *β*- 1-3 glucanase (*Bu*GH16) from *Bacteroides uniformis* JCM 13288^T^. ClustalW and ESPript 3.0 were used for sequence alignment and secondary structure information on aligned sequences, respectively. An amino acid sequence (PDB: 4BOW) of *Zobellia galactanivorans* DSM 12802, based on Phyre 2 orthologous analysis that showed 34% sequence identity, was used in this figure. Amino acid sequences of *Bacteroides uniformis* JCM 13288^T^, and *Z. galactanivorans* DSM 12802 were taken from genome sequence and NCBI respectively. The PDB-4BOW was used for deducing amino acid residues in secondary structures of *Bu*GH16.


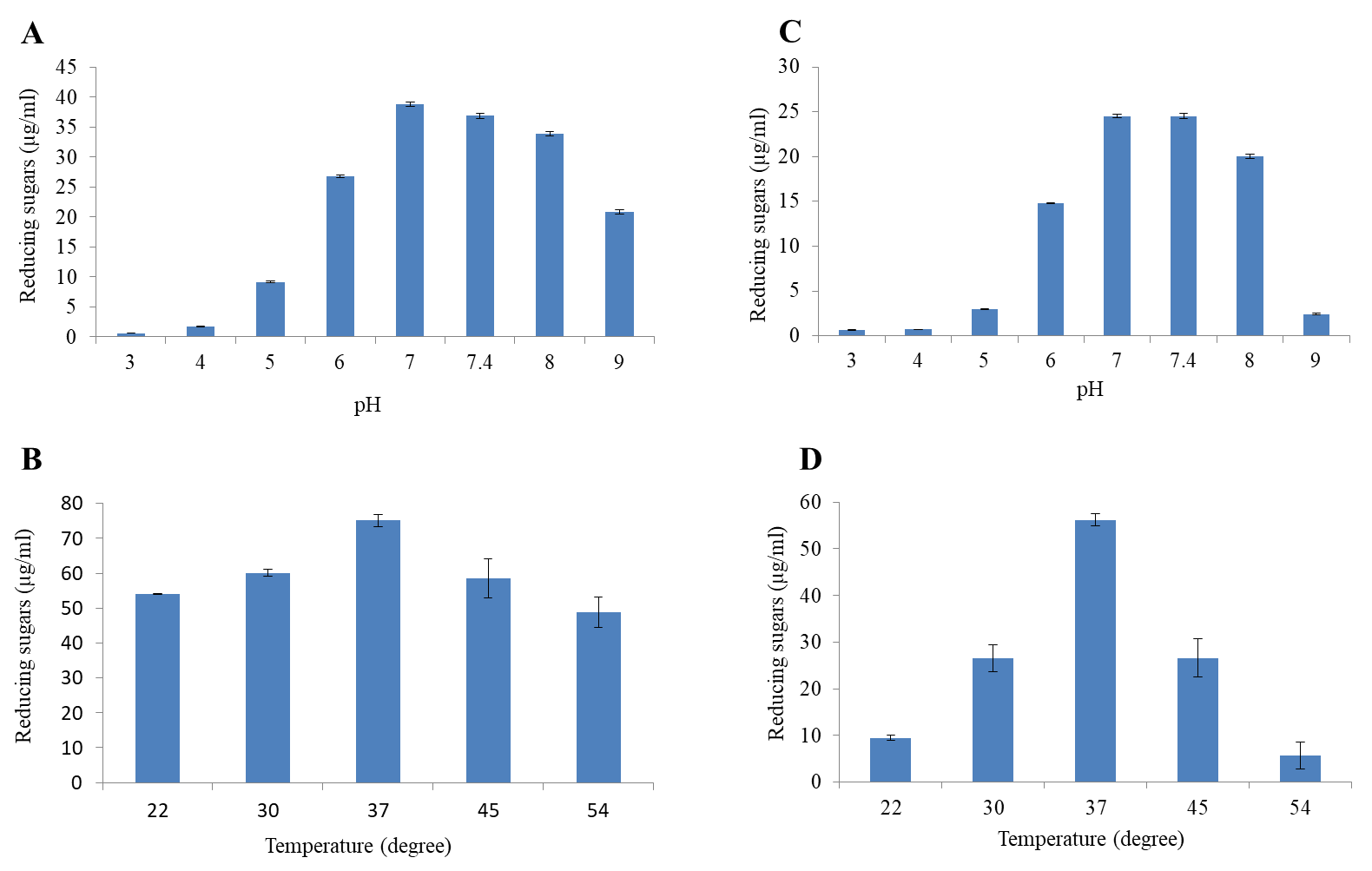


**Fig. S6.** Optimization of pH and temperature of recombinant enzymes. (A) pH profile of the *Bu*GH158, (B) temperature profile of the *Bu*GH158, (C) pH profile of the *Bu*GH30, (D) temperature profile of the *Bu*GH30. Different buffers such as phosphate – citrate (pH 3 to 5), sodium –phosphate (pH 6 and 7) and HEPES (pH to 7.4 to 9) were used at 50 mM.

**
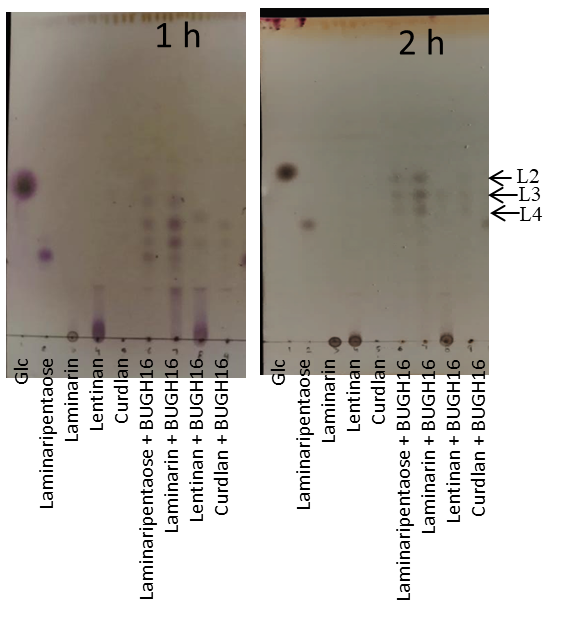
**

**Fig. S7.** Thin layer chromatography of enzymatic mixture of *Bu*GH16 with various substrates. All reactions were performed in 100 µl volume having 10 µl each of enzyme (1 mg/ml) and substrate (10 mg/ml) in 50 mM HEPES buffer at pH 7. TLC mobile phase: butanol: ethanol: water (5:3:2, v/v/v). L2- laminaribiose, L3- laminaritriose and L4- laminaritetroase.


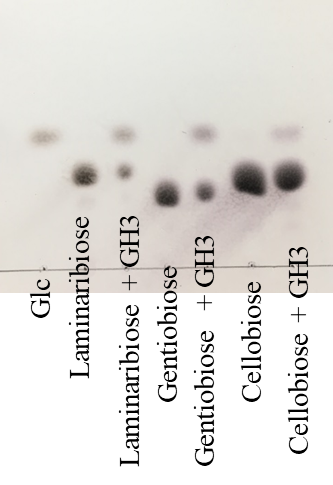


**Fig. S8.** Activity of *Bu*GH3 on laminaribiose, gentiobiose and cellobiose. The reaction was performed in 100 µl volume having 10 µl each of enzyme (1 mg/ml) and substrate (10 mg/ml) in 50 mM HEPES buffer at pH 7. TLC mobile phase: butanol: ethanol: water (5:3:2, v/v/v). Reaction of laminaribiose with *Bu*GH3 was incubated for 2 h, whereas reactions of cellobiose and gentiobiose with BuGH3 were incubated for more than 24 hours.

**
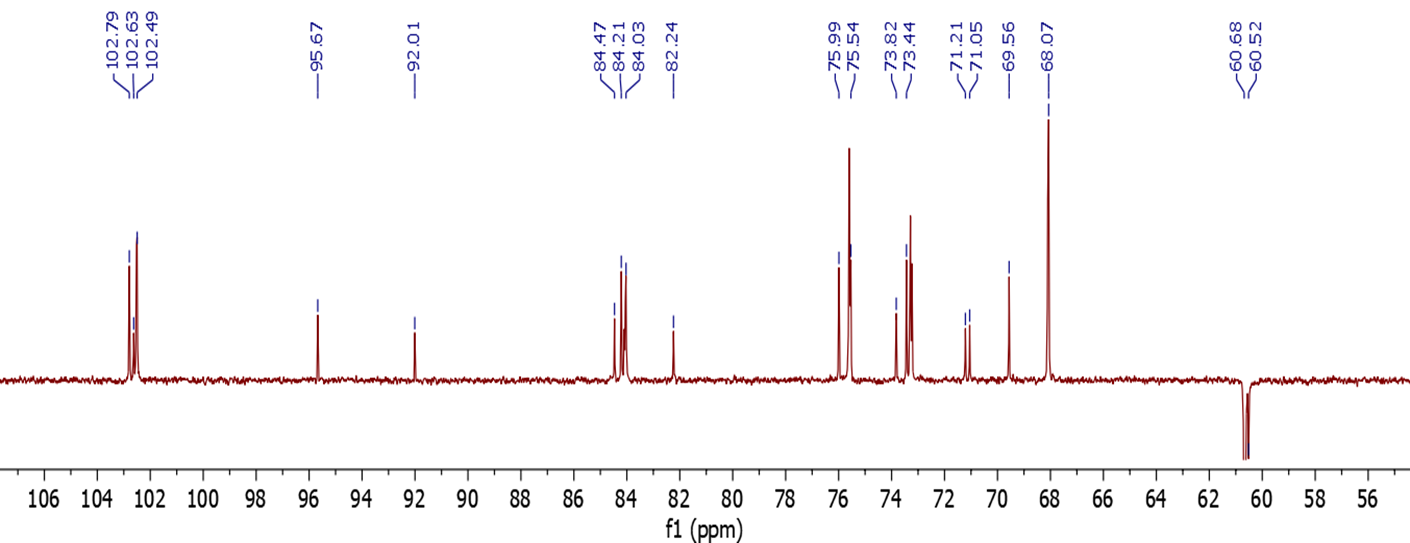
**

**Fig. S9A.** ^13^C - DEPT (135) NMR of generated *β*-1-3 linked oligosaccharides from curdlan by the action of *Bu*GH158.


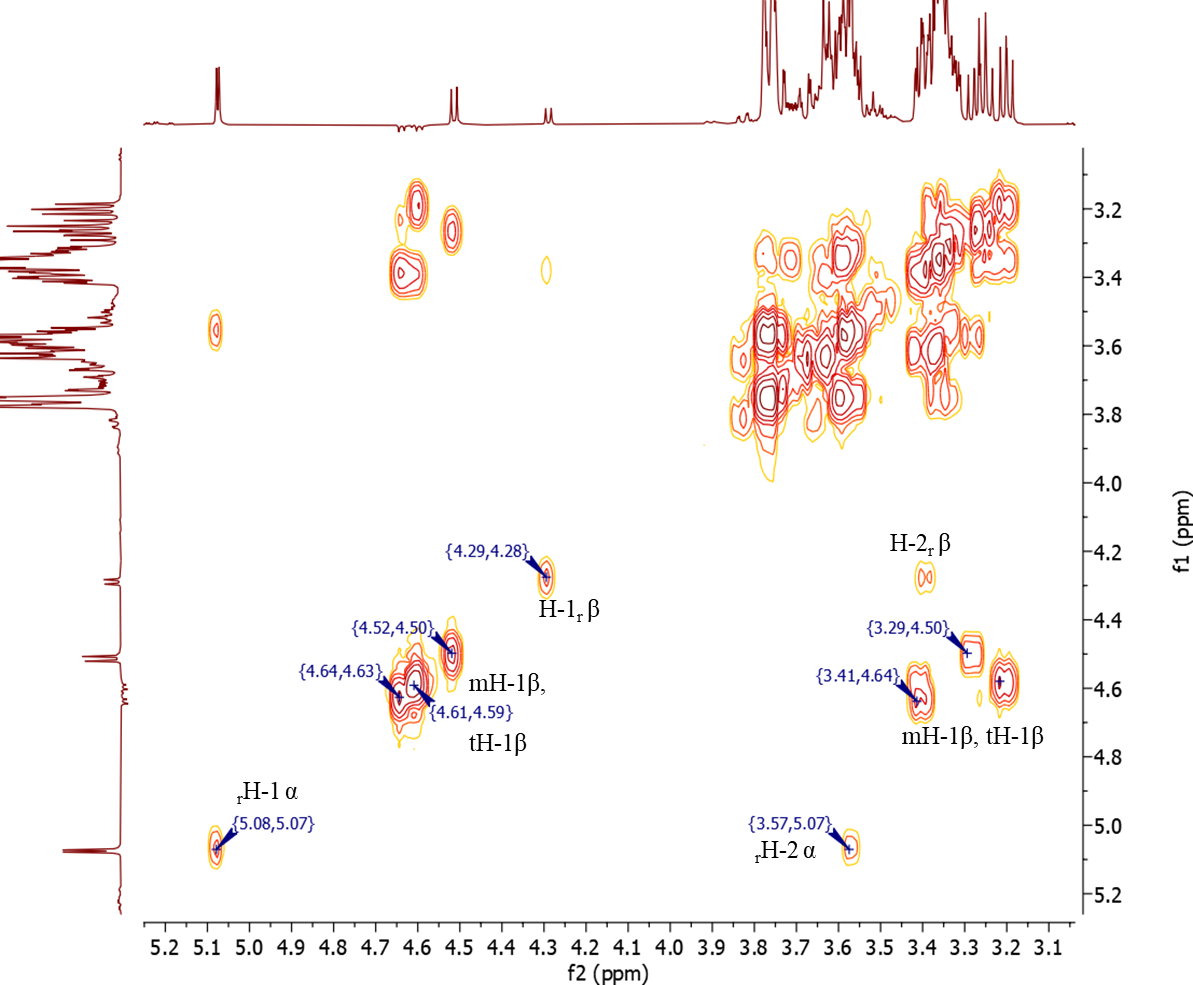


**Fig. S9B**. 2D-COSY of generated *β*-1-3 linked oligosaccharides from curdlan by the action of *Bu*GH158. *β*-d-Glc_t_ –[1-3-*β*-d-Glc_m_]_0–4_-1-3-*β*-d-Glc_r_. t-terminal, m- middle and r-reducing end sugar residues.


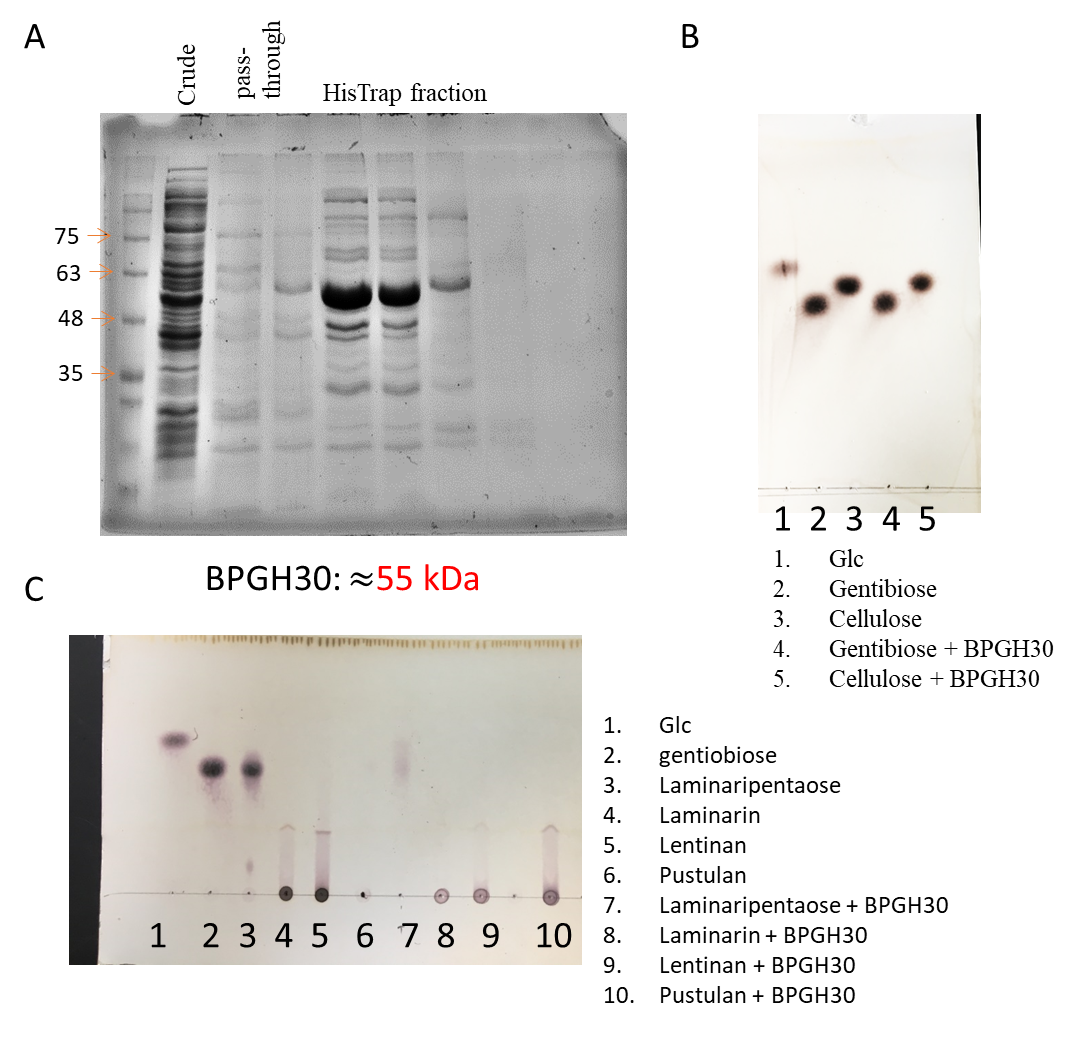


**Fig. S10**. Sodium dodecyl sulfate–polyacrylamide gel electrophoresis analysis of recombinant protein- *BP*GH30 from *Blautia producta* JCM 1471^T^ (A). Thin layer chromatography of enzymatic assays with different substrates (B and C). Enzymatic assays were performed in 100 µl reaction mixture containing 10 µl enzyme (1 mg/ml) and 10 µl of 1 % substrate in Tris-HCl buffer (pH-7.5, 50 mM) at 37 ºC for 1 h. TLC mobile phase: butanol: ethanol: water (5:3:2, v/v/v).


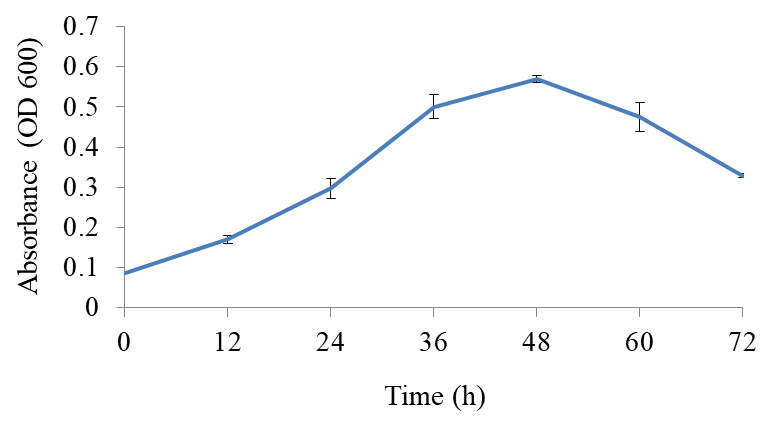


**Fig. S11** - Growth curve of *Bacteroides uniformis* JCM 13288^T^ on pustulan.


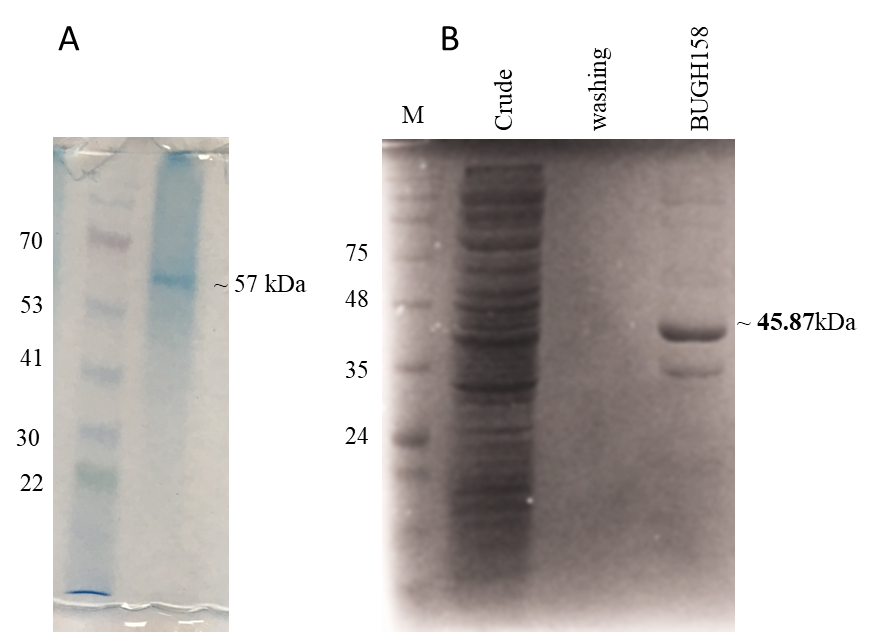


**Fig. S12.** Sodium dodecyl sulfate–polyacrylamide gel electrophoresis analysis of recombinant proteins: A – *Bu*GH30, and B- *Bu*GH158.


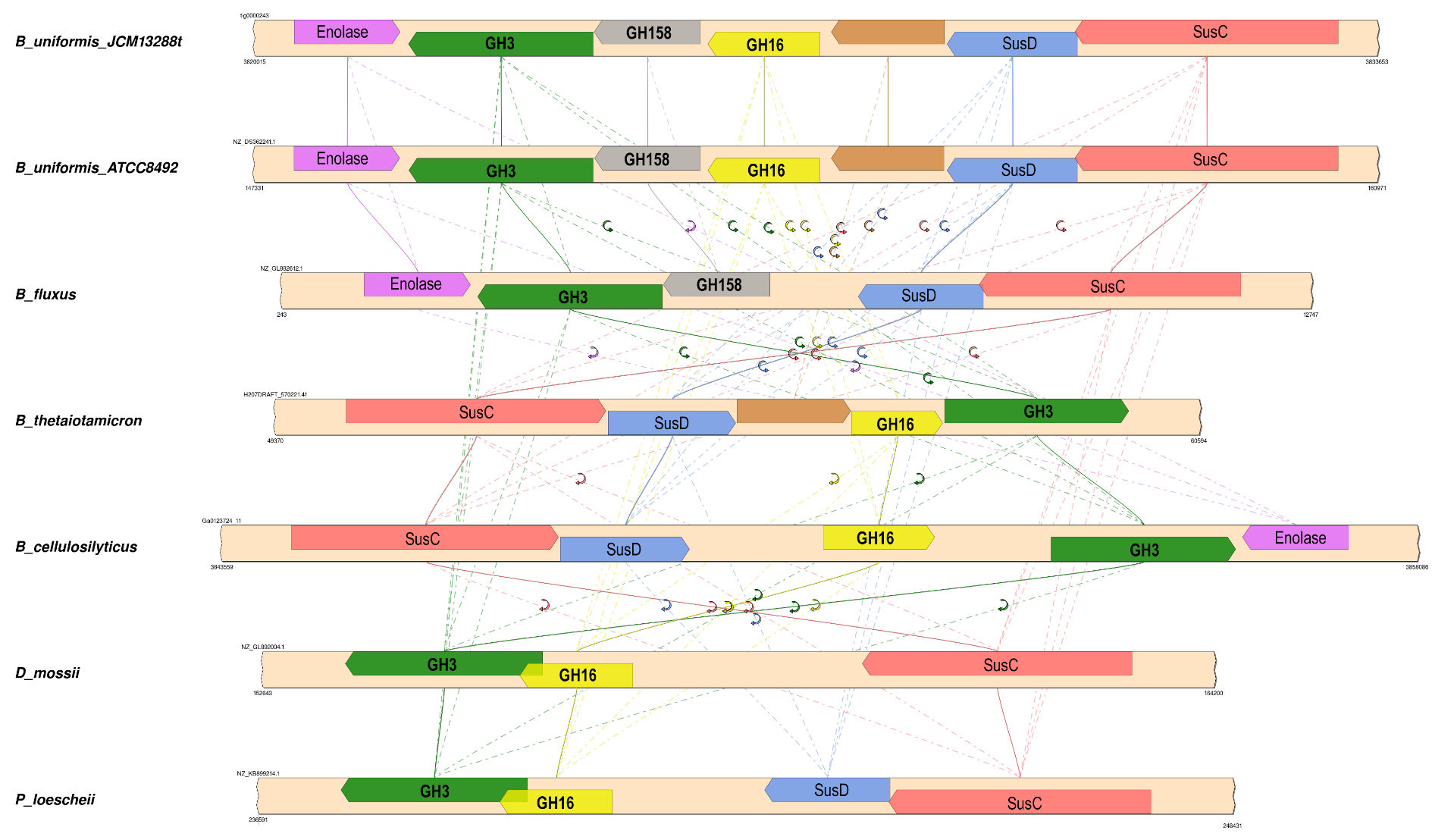


**Fig. S13: Synteny of the laminarin utilization locus among various *Bacteroidetes*.** The connecting lines show genes similarity/synteny. Arrows represent changes in gene direction. GH3 is marked in blue, GH158 in mustard yellow, and GH16 in dark green. Unlabelled proteins are hypothetical proteins. *Bacteroides uniformis* JCM 13288^T^, *B. uniformis* JCM 5828 (ATCC8492), *B. fluxus* YIT 12057, *B. cellulosilyticus* WH2, *B. thetaiotaomicron* NLAE-zl-H207, *Dysgonomonas mossii* DSM 22836 and *Prevotella loescheii* DSM 19665.

**Table S1.** List of primer used in the study

| Primer name | Sequence |
| --- | --- |
| BUGH30-F | AGACATATGCCGGAACCCAAGCCAGA |
| BUGH30-R | CTGCTCGAGTTACAACGGTAAGAAACGACTG |
| BUGH158-F | GACCATATGAGACGGTTATAAACTTTCCATTGAC |
| BUGH158-R | GAGCTCGAGTTAAAATGGAATATTTGCTGTTGAG |
| BUGH3-F | TAAAAGCTTTGCAGTCATCGAGCAGAAAAT |
| BUGH3-R | CCTCTCGAGCACCCAAAGAGCAAAGTCTCC |
| BUGH16-F | GAAAAGCTTGTGGCAGTAGCGATGACAAG |
| BUGH16-R | GGACTCGAGTTATCCACCTTCATCGTTACAGG |
| BPGH30-F | CAGAAGCTTGGATCCTGGCAGGAACAAT |
| BPGH30-R | GAACTCGAGGCATTTGAGCCATCTGTCC |

Restriction sites are underline

| Query gene ID | *Bifidobacterium adolescentis* JCM 1275^T^  Gene ID and & homology | *Bifidobacterium pseudocatenulatum*, JCM 1200^T^  Gene ID and & homology | *Ruminococcus faecis* JCM 15917  Gene ID and & homology | *Anaerostipes caccae* JCM 13470 ^T^  Gene ID and & homology | *Blautia producta*  JCM 1471^T^  Gene ID and & homology |
| --- | --- | --- | --- | --- | --- |
| BuGH_03784  (*BuGH3*) | 639764152, and 270/775 (35%) | 2563201647 and 269/775 (35%)  PDB: 5z9s (82%) | 2734498804, and 213/714 (30%),  PDB: 5WAB (43%)  2734499955 and 56/166 (34%) | NA | 2515952462,  290/751 (39%) |
| *Bu*GH_01399  (*Bu*GH30_3) | 639764163 and  120/432 (28%) | 2563201659 and 118/432 (27%) | - | NA | 2515950412, and 112/421 (27%) |
| query125423 (putative laminarin-phosphorylase) | 639763370 and  69/178 (39%) | NA | 2734500489 and 614/771 80% | NA | 2515951314, and 804/804 (100%) |
| *Bu*GH_03786, *Bu*GH16 | NA | NA | NA | NA | 2515951313 and 104/282 (37%) |

**Table S2.** Putative β1-3 utilization genes in Gram-positive bacteria (only top heat)

: No homology was found with *Bu*GH158. NA- not suitable homology found. This homology search was performed on JGI-IMG

**References**

1. Saitou N, Nei M. The neighbor-joining method: a new method for reconstructing phylogenetic trees. Molecular biology and evolution 1987; 4:406-25.

2. Felsenstein J. Confidence Limits on Phylogenies: An Approach Using the Bootstrap. Evolution; international journal of organic evolution 1985; 39:783-91.

3. Kaur G, Iyer LM, Subramanian S, Aravind L. Evolutionary convergence and divergence in archaeal chromosomal proteins and Chromo-like domains from bacteria and eukaryotes. Scientific reports 2018; 8:6196.

4. Kumar S, Stecher G, Li M, Knyaz C, Tamura K. MEGA X: Molecular Evolutionary Genetics Analysis across Computing Platforms. Molecular biology and evolution 2018; 35:1547-9.

5. Waterhouse A, Bertoni M, Bienert S, Studer G, Tauriello G, Gumienny R, et al. SWISS-MODEL: homology modelling of protein structures and complexes. Nucleic acids research 2018; 46:W296-W303.

6. Dejean G, Tamura K, Cabrera A, Jain N, Pudlo NA, Pereira G, et al. Synergy between Cell Surface Glycosidases and Glycan-Binding Proteins Dictates the Utilization of Specific Beta(1,3)-Glucans by Human Gut Bacteroides. Mbio 2020; 11.

7. Temple MJ, Cuskin F, Basle A, Hickey N, Speciale G, Williams SJ, et al. A Bacteroidetes locus dedicated to fungal 1,6-beta-glucan degradation: Unique substrate conformation drives specificity of the key endo-1,6-beta-glucanase. The Journal of biological chemistry 2017; 292:10639-50.
